# Mechanistic insights into miR-584-5p-mediated Inhibition of PDLSCs osteogenic differentiation through H2AFZ upregulation and RUNX2 suppression

**DOI:** 10.1007/s00018-025-05887-3

**Published:** 2025-10-07

**Authors:** Chengze Wang, Xiaoyan Miao, Yongzheng Li, Lingfei Ren, Bo Zheng, Zhiwei Jiang, Ying Wang, Guoli Yang

**Affiliations:** 1 Stomatology Hospital, School of Stomatology, Clinical Research Center for Oral Diseases of Zhejiang Province, Key Laboratory of Oral Biomedical Research of Zhejiang Province, Zhejiang University School of Medicine, Cancer Center of Zhejiang University, Hangzhou, 310006 China; 2 Stomatology Hospital, School of Stomatology, Zhejiang University School of Medicine, Hangzhou, 310006 China

**Keywords:** MiR-584-5p, H2AFZ, Osteogenic differentiation, Periodontal ligament stem cells, Epigenetic regulation

## Abstract

Periodontal ligament stem cells (PDLSCs) hold promise for bone regeneration, but their osteogenic differentiation is tightly regulated by various molecular mechanisms. MicroRNAs are key regulators of this process, and miR-584-5p has been identified as a potential modulator of osteogenesis. In this study, we investigated the role of miR-584-5p in the osteogenic differentiation of PDLSCs. Our findings show that overexpression of miR-584-5p inhibits osteogenic differentiation in vitro, as evidenced by reduced alkaline phosphatase activity, diminished mineralized nodule formation, and decreased expression of osteogenic markers, including ALPL, SP7, and RUNX2. In animal models, suppression of miR-584-5p enhances bone formation in both ectopic bone formation and rat calvarial defect models. Mechanistically, we demonstrate that miR-584-5p upregulates the histone variant H2AFZ, leading to its increased nuclear localization and binding to osteogenic gene promoters, including *ALPL*, *SP7*, and *RUNX2*, thereby repressing their expression. Furthermore, miR-584-5p directly targets RUNX2 mRNA, further suppressing its expression. Rescue experiments confirmed that knockdown of H2AFZ or overexpression of RUNX2 mitigates the suppressive effects of miR-584-5p on osteogenesis. Our study reveals that miR-584-5p inhibits osteogenic differentiation of PDLSCs through dual mechanisms: H2AFZ upregulation and RUNX2 suppression, offering novel insights into the epigenetic regulation of bone formation and potential therapeutic strategies for bone regeneration.

**Supplementary Information:**

The online version contains supplementary material available at 10.1007/s00018-025-05887-3.

## Introduction

Periodontal ligament stem cells (PDLSCs) play a critical role in maintaining periodontal tissue health due to their ability to differentiate into osteoblasts, facilitating the repair and regeneration of periodontal bone tissue [1–3]. The osteogenic differentiation of PDLSCs is governed by complex mechanisms, including epigenetic modifications such as microRNAs (miRNAs) and histone acetylation [4, 5]. MiRNAs regulate osteogenesis by modulating the expression of key osteogenic genes through targeting specific messenger RNAs (mRNAs) [6], while histone acetylation influences chromatin structure and promoter acetylation, thereby affecting gene transcription [7–9].

Several studies have underscored the critical role of miRNAs in regulating PDLSC osteogenic differentiation. For example, miR-21 targets Smad5, modulating osteogenesis, and inhibition of miR-21 increases alkaline phosphatase (ALP) activity and mineralization levels [10]. Liao et al. demonstrated that the long non-coding RNA LINC00968 promotes osteogenic differentiation through the miR-3658/RUNX2 axis, providing new insights into dental pulp stem cell differentiation [11]. Additionally, overexpression of miR-143-3p or silencing of Krüppel-like factor 5 (KLF5) suppresses osteogenic marker expression and mineralized nodule formation in PDLSCs by inactivating the Wnt/β-catenin pathway, an effect that can be reversed by pathway activation [12]. In our previous study, bioinformatics analysis identified miR-584-5p as a potential regulator of osteogenic differentiation, although the precise mechanisms remain unclear [13]. Histone modifications also significantly impact PDLSC osteogenesis. PDLSCs derived from periodontitis patients show impaired differentiation, linked to downregulation of histone acetyltransferase (HAT) GCN5. GCN5 deficiency reduces osteogenesis by regulating DKK1 expression through histone H3 lysine acetylation [14]. Additionally, decreased levels of GCN5 and MORF under periodontitis conditions inhibit PDLSC differentiation into osteoblasts [15]. Li et al. showed that enhancing HAT levels can reverse impaired osteogenic differentiation, highlighting the inhibitory role of histone deacetylase 9 (HDAC9) [16].

H2AFZ, also known as H2A.Z, is a variant of histone H2A and a crucial component of chromatin [17]. It replaces standard H2A in nucleosomes, influencing chromatin structure and gene regulation at promoters and enhancers [18]. While H2A.Z is implicated in various biological processes and diseases, its role in osteogenic differentiation remains unexplored.

In this study, we investigate the role of miR-584-5p in regulating osteogenesis in PDLSCs. Our results show that miR-584-5p negatively modulates the enrichment of acetylated H2AFZ (acH2AFZ) at the promoters of key osteogenic genes, including RUNX2, SP7, and ALPL, leading to reduced transcription of these genes. Furthermore, we demonstrate that miR-584-5p directly interacts with RUNX2, inhibiting its expression and impairing osteogenic differentiation and new bone formation in PDLSCs.

## Materials and methods

### Antibodies and reagents

Rabbit antibodies against RUNX2 (ab92336), ALPL (ab305305), and SP7 (ab209484) were purchased from Abcam Inc. Rabbit antibodies against H2AFZ (#50722) and acetylated H2AFZ (H2AFZac, K4/K7/K11) (#75336) were obtained from Cell Signaling Technology. Rabbit-derived HDAC1 antibody (10197-1-AP) was from Proteintech (Wuhan, China). GAPDH and H3 antibodies were procured from Boster Biological Technology (Wuhan, China). Goat anti-rabbit and goat anti-mouse HRP-conjugated secondary antibodies were sourced from Cell Signaling Technology (Beverly, MA, USA). Alexa Fluor 488 and 594-conjugated secondary antibodies were purchased from Invitrogen. The ChIP assay kit was acquired from ABclonal. Dexamethasone, ascorbic acid, and β-glycerophosphate were obtained from Sigma-Aldrich Corporation (St. Louis, MO, USA).

### Isolation and characterization of human PDLSCs

The study was approved by the Institutional Animal Care and Use Committee of Zhejiang University, with written informed consent from all patients. PDLSCs were isolated from three healthy human premolars extracted for orthodontic reasons. Periodontal ligament tissue was gently scraped from the middle third of the root surfaces and processed as previously described [13, 18, 19]. Individual cell colonies were isolated, trypsinized, and expanded. We performed multi-lineage differentiation characterization of the isolated PDLSCs (Fig. S5). All experiments were independently repeated at least three times.

### Cell culture and transfection

Based on the effects of different concentrations of miR-584-5p inhibitor on PDLSC proliferation and ALP activity, we selected 25 nM miR-584-5p inhibitor for subsequent experiments (Fig. S4B-D). For miRNA transfection, cells were transfected with miR-584-5p mimics or inhibitors at 25 nM using jetPRIME^®^ transfection reagent at a 1:2 ratio (miRNA: transfection reagent), following the manufacturer’s protocol. For H2AFZ knockdown, PDLSCs were transfected with 25 nM siRNA targeting H2AFZ or negative control siRNA using the same transfection reagent. The sequences of the miRNAs and siRNAs are provided in Table S1. For RUNX2 overexpression, PDLSCs were transduced with lentiviral particles carrying RUNX2 or control lentivirus at an MOI of 30, with 8 µg/ml polybrene to enhance transduction efficiency. After 3 days, puromycin selection (1 µg/ml) was applied for 7 days to establish stable overexpression.

### ALP and Alizarin red staining assays

PDLSCs were seeded into 12-well plates at densities of 1.5 × 10^5^ cells/well for ALP staining and 1.5 × 10^5^ cells/well for Alizarin Red staining. Cells were cultured in osteogenic differentiation medium (ODM) consisting of α-MEM supplemented with 10% FBS, 50 µM ascorbic acid, 10 µM dexamethasone, and 10 mM β-glycerophosphate. ALP activity was measured using an Alkaline Phosphatase Activity Detection Kit (Yeasen, China). ALP staining was performed using an alkaline phosphatase kit (Beyotime, China), and Alizarin Red staining was conducted with a 2% Alizarin Red S solution (ScienCell, USA). Calcium deposition was quantified by dissolving the mineralized matrix in 10% cetylpyridinium chloride and measuring absorbance at 562 nm.

### Protein extraction and Western blot analysis

Cells were lysed in RIPA buffer on ice for 30 min. Lysates were centrifuged at 12 000 rpm for 30 min at 4 °C to collect supernatants. Protein samples (20 µg) were separated on 10% SDS-PAGE gels and transferred to PVDF membranes. Membranes were blocked with TBST containing 5% non-fat dry milk for 1 h at room temperature, then incubated overnight at 4 °C with primary antibodies against RUNX2, SP7, ALPL, H2AFZ, and GAPDH at specified dilutions. After washing, membranes were incubated with HRP-conjugated secondary antibodies (1:10 000) for 1 h. Bands were visualized using an enhanced chemiluminescence system (Bio-Rad, USA) and quantified using ImageJ software.

### Co-immunoprecipitation

PDLSCs were seeded in 10 cm dishes and subjected to osteogenic induction for 3 days. Cells were harvested and lysed in co-IP buffer. For co-immunoprecipitation, cell lysates were incubated with either HDAC1 antibody or H2AFZ antibody overnight at 4 °C, followed by incubation with magnetic beads for 2 h at 4 °C with rotation. The immunocomplexes were captured using a magnetic separator and washed three times with wash buffer. Bound proteins were eluted by boiling in SDS loading buffer and subjected to standard Western blot analysis to detect protein-protein interactions between HDAC1 and H2AFZ during early osteogenic differentiation.

### RNA isolation and real-time qPCR analysis

Total RNA was extracted using Trizol^®^ reagent per the manufacturer’s instructions and reverse-transcribed into cDNA. Quantitative real-time PCR (RT-qPCR) was performed with SYBR^®^ Premix Ex Taq™ on a Bio-Rad CFX96 system. Each sample was analyzed in triplicate, and experiments were repeated independently three times. Relative gene expression was calculated using the 2^−ΔΔCT^ method. Primer sequences are listed in Table S2.

### Immunofluorescence assay

Glass coverslips (8 mm diameter) were placed into 48-well plates, and PDLSCs were seeded at 3 × 10^3^ cells per well, cultured for three days. Cells were fixed, washed with PBS, and blocked with 2% bovine serum albumin at room temperature for 30 min. Cells were incubated overnight at 4 °C with primary antibody against H2AFZ (1:200 dilution). After washing, cells were incubated with an Alexa Fluor 488-conjugated secondary antibody for 1 h at room temperature. Nuclei were stained with DAPI. For tissue samples from in vivo experiments, standard procedures of sectioning, deparaffinization, and rehydration were performed. Since both SP7 and H2AFZ primary antibodies are rabbit-derived, tyramide signal amplification (TSA) technology was employed to avoid cross-reactivity and enable simultaneous detection.

### ChIP-qPCR

PDLSCs 1 × 10^7^ cells were cultured under specific conditions. ChIP assays were conducted to investigate H2AFZ and acH2AFZ binding to genomic regions. Cells were cross-linked with 1% formaldehyde for 10 min, quenched with glycine for 5 min, harvested, lysed, and sonicated to fragment chromatin to 200–500 bp. Chromatin was immunoprecipitated overnight at 4 °C with antibodies specific to H2AFZ and acH2AFZ. Protein-DNA complexes were captured using protein A/G agarose beads, eluted, and reverse cross-linked. DNA was purified and analyzed by qPCR using specific primers (Table S2). Relative enrichment was calculated using the 2^−ΔΔCT^ method.

### Animal models

All animal experiments were conducted in accordance with the guidelines set by the Institutional Animal Care and Use Committee of Zhejiang University (No. ZJU20210530). For the rat cranial defect model, 48 male Sprague-Dawley rats (age: 6–8 weeks, body Weight: 200–250 g) were randomly divided into four groups: miR-584-5p inhibitor, NC inhibitor, siNC, and siH2AFZ. Each group contained 12 animals. Bilateral cranial defects (5 mm diameter) were created using a trephine drill at 800 rpm with saline irrigation [20, 21]. Collagen scaffolds containing transfected PDLSCs were placed in defects. Surgical incisions were closed with absorbable sutures. Animals were housed under stable conditions and euthanized at 4 and 8 weeks post-operation for sample collection and micro-CT analysis.

For the subcutaneous ectopic ossification model, 20 BALB/c nude mice received implants of bone-inductive calcium phosphate bioceramics seeded with transfected PDLSCs. After incubation in osteogenic induction medium for 7 days, scaffolds were implanted subcutaneously. Mice were monitored postoperatively, and implants were harvested at 8 weeks for histological and micro-CT analysis.

### Micro-CT analysis

Excised tissues were fixed and scanned using high-resolution micro-CT (Skyscan1176). Data were reconstructed and analyzed using software such as NRecon, CTAn, Dataviewer, and Mimics for three-dimensional reconstruction and morphological analysis.

For rat cranial defect samples, images were reoriented using Dataviewer to align coronal and horizontal planes perpendicular to the trephine drill direction. Images were exported and analyzed in CTAn. A circular region of interest (ROI) with a 5 mm diameter was defined at the defect site. Density thresholds from 152 to 210 were set to include bone tissue, and bone parameters were calculated.

### Immunohistochemistry

Fixed tissues were dehydrated, cleared in xylene, and embedded in paraffin. Sections were deparaffinized, rehydrated, and antigen retrieval was performed by heating. Sections were outlined with a hydrophobic barrier pen and blocked with goat serum. They were incubated with specific primary antibodies at appropriate dilutions, followed by species-specific HRP-conjugated secondary antibodies. Visualization was achieved using DAB chromogen, resulting in a brown precipitate at antigen sites. Nuclei were counterstained to enhance contrast. Sections were dehydrated, cleared, and mounted using neutral resin. Images were captured using a light microscope.

### Luciferase reporter assay

 293 T cells were seeded in 12-well plates at 2.5 × 10^5^ cells/well and culture

d for 24 h. For miR-584-5p targeting validation, cells were co-transfected with 1.0 µg RUNX2 3’UTR dual-luciferase reporter plasmid and 6 µL miR-584-5p mimic (20 µM) or negative control using jetPrime transfection reagent (Table S3). Cells were harvested at 24 and 48 h for luciferase analysis (Dual Luciferase Reporter Gene Assay Kit, Yeasen, Shanghai, China).

For promoter activity analysis, three dual-luciferase reporter plasmids were constructed: pALPL-FLuc-SV40-hRLuc, pRUNX2-FLuc-SV40-hRLuc, and pSP7-FLuc-SV40-hRLuc (Table S3). Cells were co-transfected with each reporter plasmid (1.0 µg) and either pCDH-hRUNX2, pCDH-hSP7, or pCDH-EGFP control vector (1.0 µg each). Transfection mixtures were prepared in 500 µL serum-free DMEM with jetPrime reagent, incubated for 20 min at room temperature, then added to cells. After 5 h, medium was replaced with complete DMEM. Cells were harvested 48 h post-transfection for dual-luciferase analysis.

### Statistical analysis

Statistical analysis was performed using GraphPad Prism 6.0 (GraphPad Software, USA). The data were analyzed using Student’s t-test for comparisons between two groups, and one-way ANOVA followed by Tukey’s post hoc test for multiple comparisons. A P-value less than 0.05 was considered statistically significant.

## Results

### MiR-584-5p Inhibits Osteogenic Differentiation of PDLSCs In Vitro

**Fig. 1:**
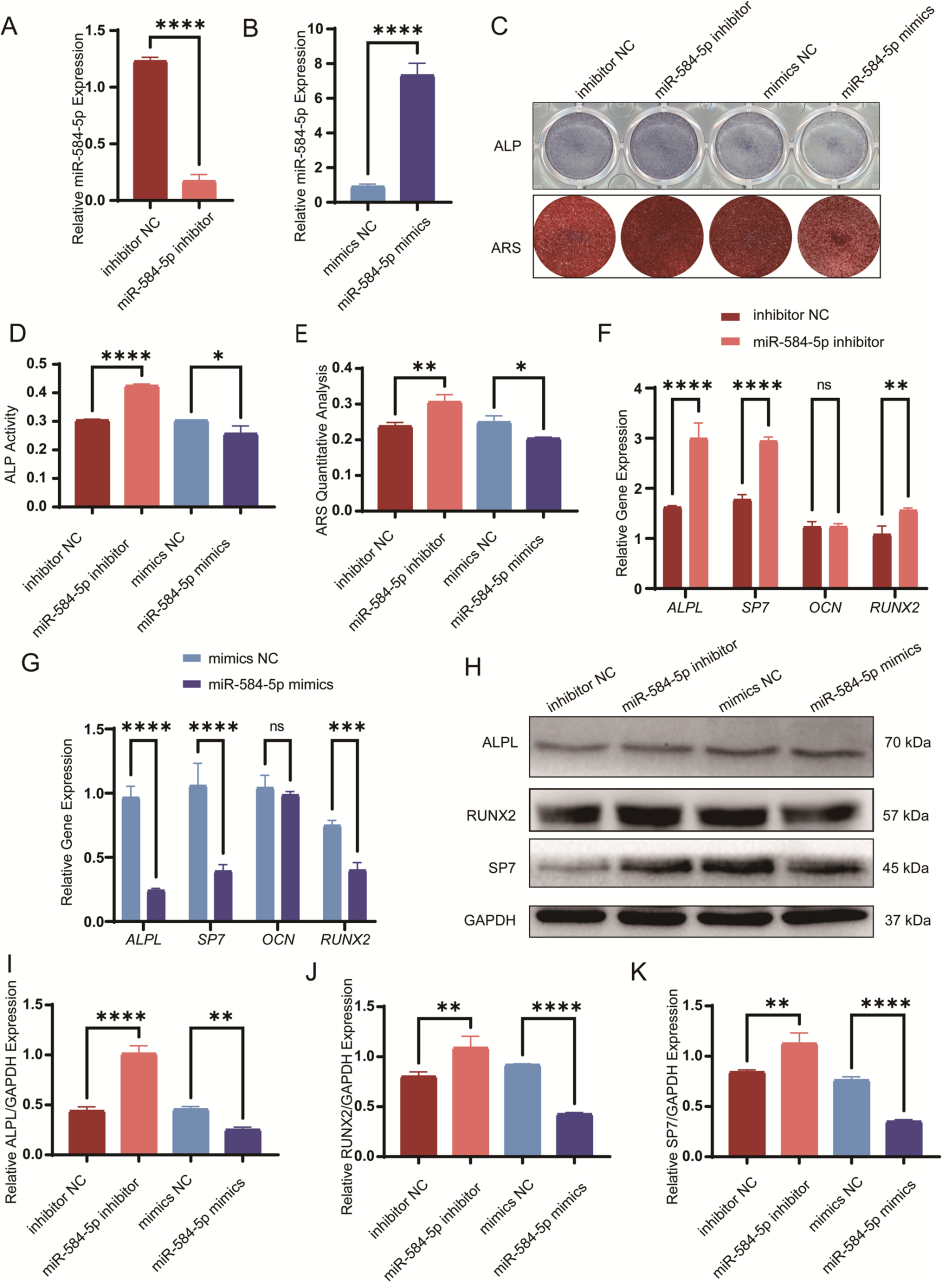
miR-584-5p inhibits osteogenic differentiation of PDLSCs in vitro. **A**, **B** Quantitative PCR (qPCR) analysis of miR-584-5p expression in PDLSCs after transfection with miR-584-5p mimics or inhibitors, confirming overexpression and knockdown efficiency. **C **ALP staining on day 7 and ARS staining on day 14 of osteogenic induction in PDLSCs transfected with miR-584-5p mimics or inhibitors; representative images are shown. **D**, **E **Quantitative analysis of ALP activity (**D**) and ARS staining (**E**) demonstrating decreased osteogenic differentiation with miR-584-5p overexpression and enhanced differentiation upon inhibition. **F**, **G **qPCR analysis of mRNA levels of osteogenic markers ALPL, SP7, OCN, and RUNX2 in PDLSCs after modulation of miR-584-5p expression. **H**–**K **Western blot analysis (**H**) and quantification (**I–K**) of osteogenic proteins ALPL, RUNX2, and SP7, showing reduced protein levels with miR-584-5p overexpression and increased levels upon inhibition. Data are presented as mean ± SD from at least three independent experiments; *p < 0.05, **p < 0.01, ***p < 0.001, ****p < 0.0001; ns nonsignificant

We investigated miR-584-5p’s role in the osteogenic differentiation of PDLSCs by modulating its expression with specific mimics and inhibitors. Quantitative PCR (qPCR) confirmed significant overexpression or inhibition of miR-584-5p in transfected cells (Fig. 1 A-B). Overexpression resulted in reduced ALP staining and decreased mineralized nodule formation, whereas inhibition enhanced these indicators of osteogenic differentiation (Fig. 1 C-E). Expression levels of osteogenic markers—ALPL, SP7, and RUNX2—were decreased at both mRNA and protein levels upon miR-584-5p overexpression and increased upon its inhibition (Fig. 1 F-K). These results indicate that miR-584-5p functions as a negative regulator of osteogenic differentiation in PDLSCs in vitro.

**Fig. 2:**
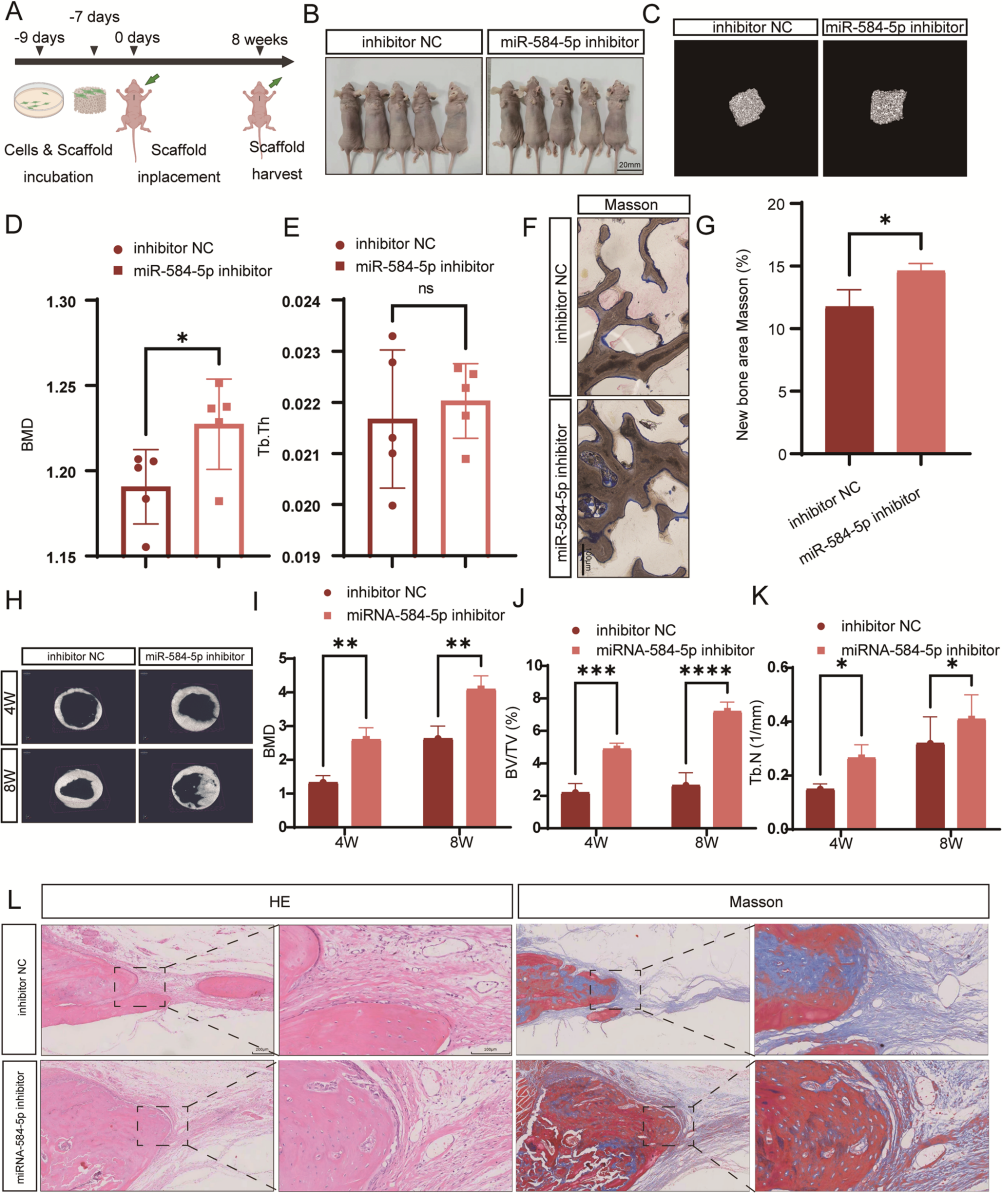
miR-584-5p inhibition promotes bone formation in vivo. **A** Schematic diagram of the ectopic bone formation model: PDLSCs transfected with miR-584-5p inhibitors or negative control were implanted subcutaneously into nude mice. **B** The nude mouse model images are shown (scale bar: 20 mm). Micro-CT images showing increased BMD in the miR-584-5p inhibitor group compared to controls. **C–E** Quantitative analysis of new bone volume (**C**), BMD (**D**), and trabecular thickness (**E**) from micro-CT data. **F**,** G** Masson’s trichrome staining of tissue sections displaying enhanced collagen deposition and new bone formation in the inhibitor group (scale bar: 100 μm). **H** Micro-CT images show three-dimensional reconstructed images of the rat calvarial defect models in different groups. **I** Micro-CT images analysis illustrating increased new bone formation in the inhibitor-treated group. **J**, **K** Quantitative analysis of bone volume fraction (**J**) and trabecular number (**K**) in calvarial defects. **L** Hematoxylin and eosin (HE) and Masson’s trichrome staining of calvarial sections showing more mature bone structures in the inhibitor group. Data represent mean ± SD; *p < 0.05, **p < 0.01, ***p < 0.001, ****p < 0.0001; ns nonsignificant

Figure 2 miR-584-5p inhibits osteogenic differentiation of PDLSCs in vitro. **A**,** B** Quantitative PCR (qPCR) analysis of miR-584-5p expression in PDLSCs after transfection with miR-584-5p mimics or inhibitors, confirming overexpression and knockdown efficiency. **C** ALP staining on day 7 and ARS staining on day 14 of osteogenic induction in PDLSCs transfected with miR-584-5p mimics or inhibitors; representative images are shown. **D**,** E** Quantitative analysis of ALP activity (**D**) and ARS staining (**E**) demonstrating decreased osteogenic differentiation with miR-584-5p overexpression and enhanced differentiation upon inhibition. **F**,** G** qPCR analysis of mRNA levels of osteogenic markers *ALPL*, *SP7*, *OCN*, and *RUNX2* in PDLSCs after modulation of miR-584-5p expression. **H–K** Western blot analysis (**H**) and quantification (**I–K**) of osteogenic proteins ALPL, RUNX2, and SP7, showing reduced protein levels with miR-584-5p overexpression and increased levels upon inhibition. Data are presented as mean ± SD from at least three independent experiments; *p < 0.05, **p < 0.01, ***p < 0.001, ****p < 0.0001; ns nonsignificant.

### Inhibition of miR-584 Expression Promotes New Bone Formation In Vivo

To validate our in vitro findings, we employed ectopic bone formation and rat calvarial defect models (Fig. 2A-B). In the ectopic model, PDLSCs transfected with miR-584-5p inhibitors formed more bone tissue, evidenced by higher bone mineral density (BMD) in micro-CT scans and increased collagen deposition in Masson-stained sections (Fig. 2C-G). In the calvarial defect model, local administration of PDLSCs with miR-584-5p inhibitors significantly enhanced bone regeneration within critical-sized defects (Fig. 2H). Micro-CT imaging showed increased new bone formation, and quantitative analysis demonstrated higher new bone volume and BMD in the inhibitor-treated group compared to controls (Fig. 2I-L). These in vivo results confirm that suppression of miR-584-5p promotes bone regeneration, aligning with our in vitro observations.

### MiR-584-5p upregulates H2AFZ expression and nuclear localization

**Fig. 3:**
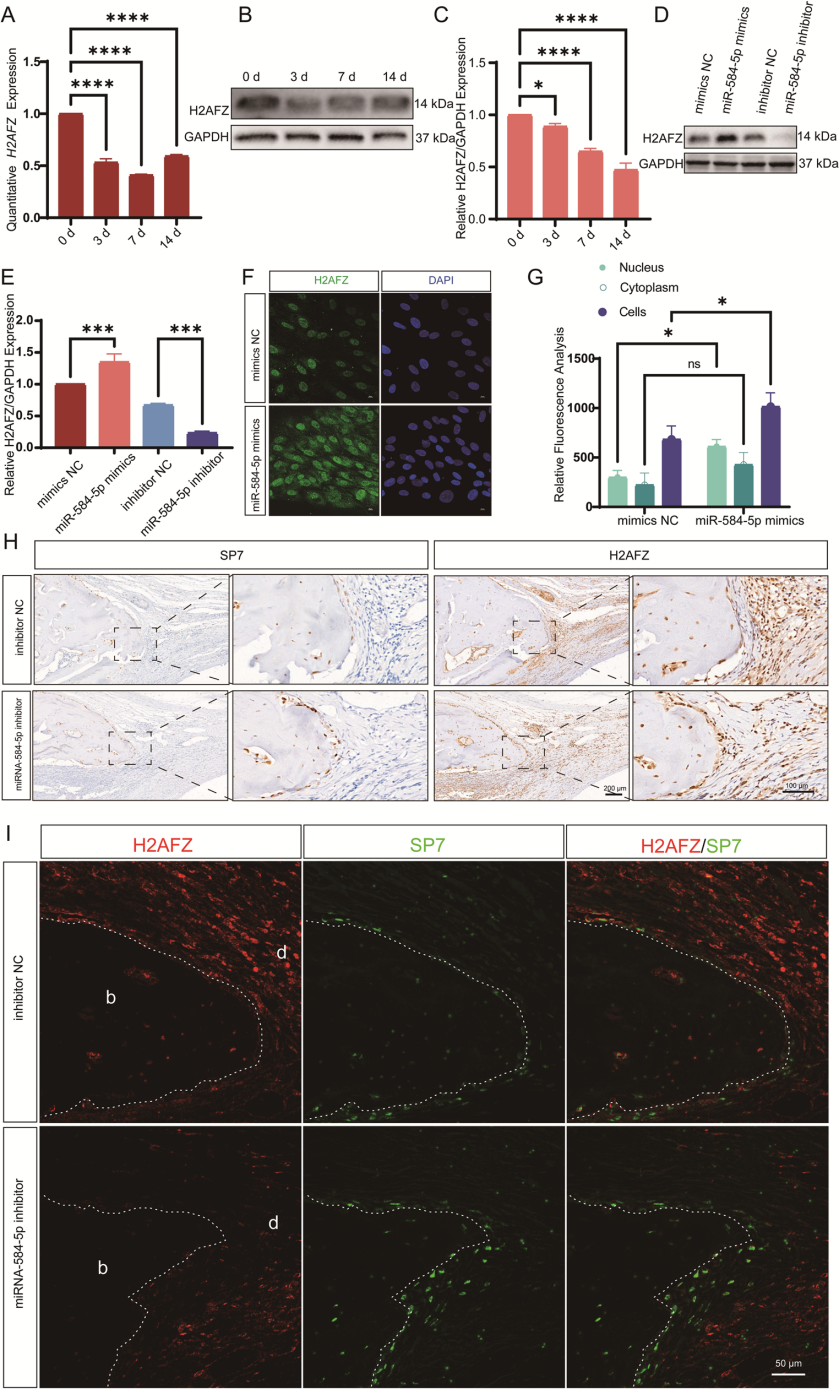
miR-584-5p regulates H2AFZ expression during osteogenic differentiation. **A–C** qPCR (**A**) and Western blot (**B**) analyses showing that H2AFZ mRNA and protein levels decrease during osteogenic induction of PDLSCs over time (**C**). **D**, **E** Western blot analysis (**D**) and quantification (**E**) indicating that miR-584-5p overexpression increases H2AFZ protein levels, while inhibition decreases them. **F**, **G** Immunofluorescence staining (**F**) and quantitative fluorescence intensity analysis (**G**) demonstrating enhanced nuclear localization of H2AFZ in PDLSCs transfected with miR-584-5p mimics compared to controls (scale bar: 10 μm). **H**, **I** Immunohistochemistry (**H**) and immunofluorescence staining (**I**) of calvarial defect tissues showing increased expression of the osteogenic marker SP7 and reduced H2AFZ expression in the miR-584-5p inhibitor group versus control. *p < 0.05, ***p < 0.001, ****p < 0.0001; ns nonsignificant

Using miRNA target gene prediction databases, we identified potential interactions between miR-584-5p and both H2AFZ and RUNX2 (Fig. S3). Considering miRNAs often regulate downstream genes, we examined the relationship between miR-584-5p and the histone variant H2AFZ (H2A.Z). QPCR and Western blot analyses showed that H2AFZ mRNA and protein levels decreased during osteogenic induction of PDLSCs (Fig. 3A-C). Overexpression of miR-584-5p increased H2AFZ expression, while its inhibition led to decreased H2AFZ levels (Fig. 3D-E). Immunofluorescence staining revealed enhanced nuclear localization of H2AFZ in cells overexpressing miR-584-5p compared to controls (Fig. 3F-G). In vivo, the miR-584-5p inhibitor group exhibited increased expression of the osteogenic marker SP7 and reduced H2AFZ expression in calvarial defect tissues (Fig. 3H-I). These findings suggest that miR-584-5p upregulates H2AFZ expression and promotes its nuclear accumulation, influencing osteogenic differentiation.

### H2AFZ mediates miR-584-5p-induced inhibition of osteogenic differentiation

**Fig. 4:**
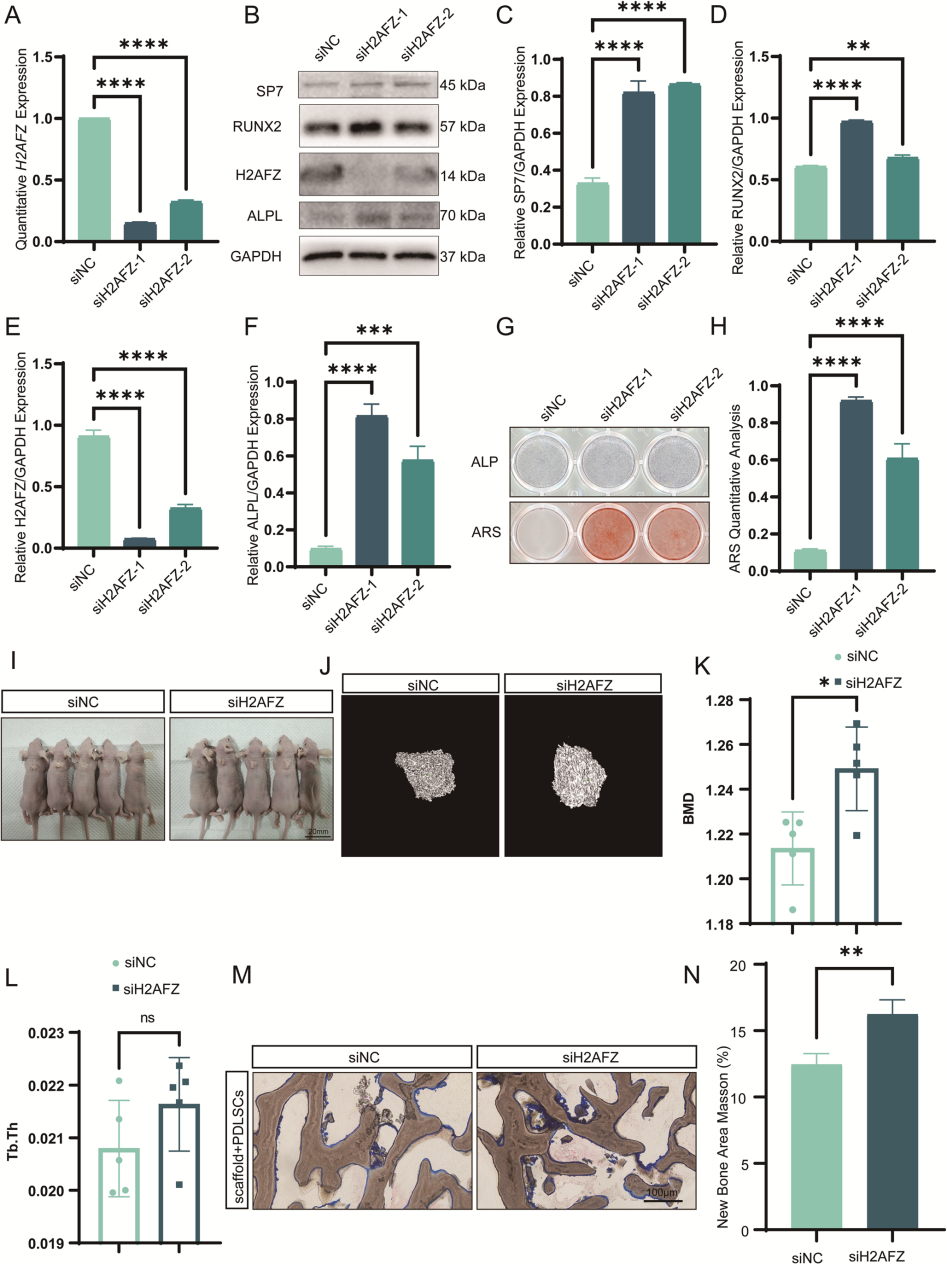
Knockdown of H2AFZ enhances osteogenic differentiation of PDLSCs. **A**,** B** qPCR (**A**) and Western blot (**B**) analyses confirming efficient knockdown of H2AFZ mRNA and protein levels in PDLSCs using two specific siRNAs. **C**–**F** Western blot analysis (**C**) and quantification (**D**–**F**) showing that H2AFZ knockdown leads to upregulation of osteogenic proteins SP7, RUNX2, and ALPL, with greater effects observed with lower H2AFZ expression. **G** ALP staining on day 7 and ARS staining on day 14 indicating enhanced osteogenic differentiation upon H2AFZ knockdown. **H** Quantitative analysis of ARS staining confirming increased osteogenesis. **I**–**N** In a subcutaneous ectopic bone formation model (**I**), micro-CT images (**J**), quantitative analysis of bone parameters (**K**–**L**), and Masson’s trichrome staining and analysis (**M**, **N**) show that H2AFZ-silenced PDLSCs promote bone formation and collagen deposition (scale bar: 100 μm). *p < 0.05, **p < 0.01, ***p < 0.001, ****p < 0.0001; ns nonsignificant

To determine H2AFZ’s role in osteogenesis, we knocked down its expression using specific siRNAs, which effectively reduced H2AFZ mRNA and protein levels (Fig. 4A-B). H2AFZ knockdown led to significant upregulation of osteogenic markers SP7, RUNX2, and ALPL (Fig. 4C-F). ALP and ARS staining confirmed that silencing H2AFZ enhanced ALP activity and promoted mineralized nodule formation (Fig. 4G-H). In vivo, PDLSCs with H2AFZ knockdown demonstrated increased BMD, collagen deposition, and new bone formation in both ectopic bone formation (Fig. 4I-N) and calvarial defect models (Fig. S1), promoting bone repair.

**Fig. 5:**
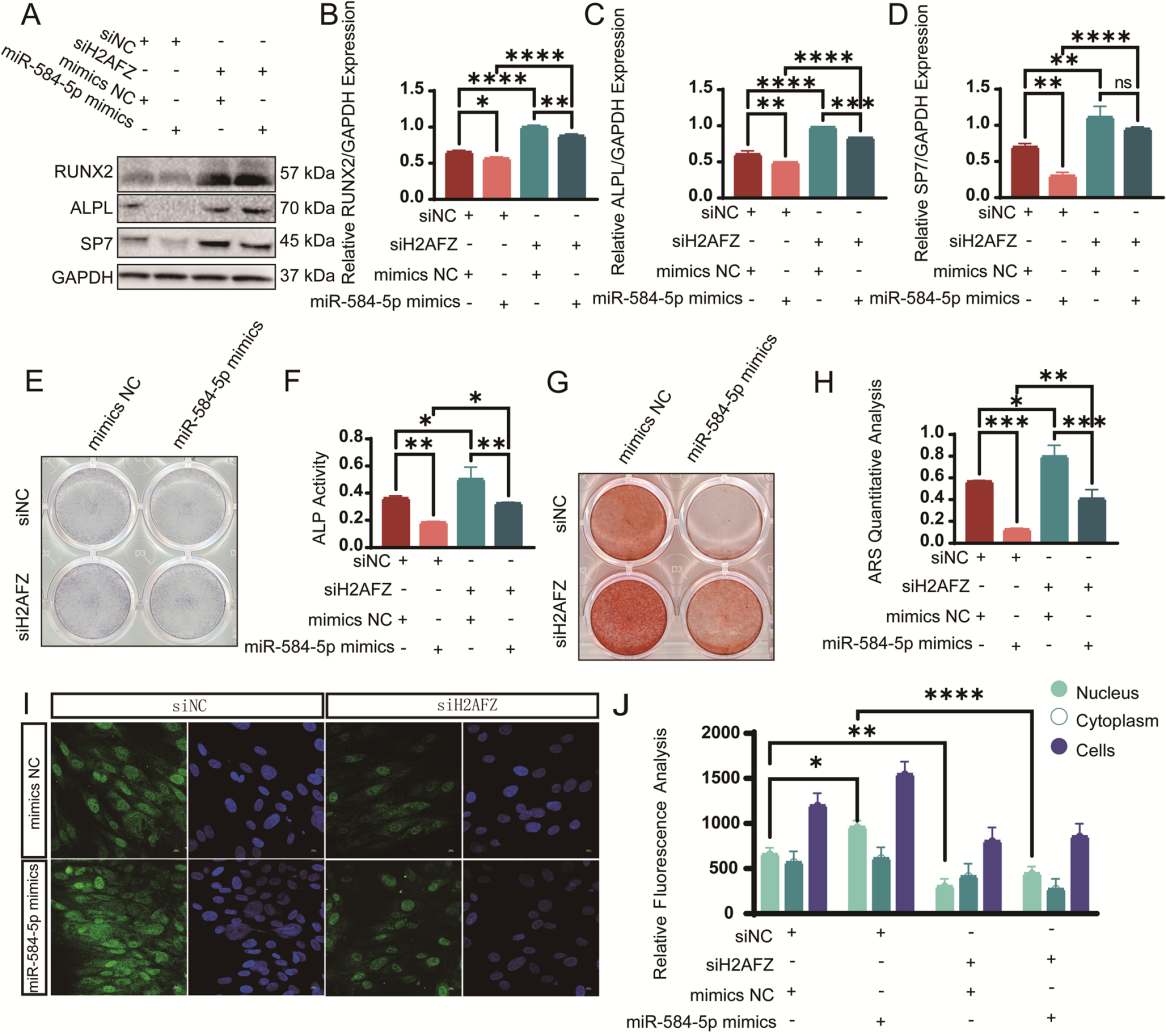
H2AFZ knockdown rescues the inhibitory effects of miR-584-5p on osteogenesis. **A**–**D** Western blot analysis (**A**) and quantification (**B**–**D**) demonstrating that knockdown of H2AFZ restores the expression of osteogenic proteins RUNX2, ALPL, and SP7 in PDLSCs overexpressing miR-584-5p. **E**, **F** ALP staining (**E**) and ALP activity assay (**F**) indicating that H2AFZ knockdown reverses the decreased osteogenic activity caused by miR-584-5p overexpression. **G**, **H** ARS staining (**G**) and quantification (**H**) showing restoration of mineralization upon H2AFZ knockdown despite elevated miR-584-5p levels. **I**, **J** Immunofluorescence staining (**I**) and fluorescence intensity analysis (**J**) confirming reduced nuclear accumulation of H2AFZ when knocked down in the presence of miR-584-5p overexpression (scale bar: 10 μm). *p < 0.05, **p < 0.01, ***p < 0.001, ****p < 0.0001; ns nonsignificant

Rescue experiments showed that H2AFZ knockdown restored the expression levels of osteogenic proteins RUNX2, ALPL, and SP7 in miR-584-5p overexpressing cells (Fig. 5A-D). Functionally, ALP activity and mineralization were also restored upon H2AFZ knockdown in the presence of elevated miR-584-5p levels (Fig. 5E-H). Immunofluorescence confirmed that H2AFZ nuclear accumulation induced by miR-584-5p overexpression was reduced when H2AFZ was knocked down (Fig. 5I-J).

**Fig. 6:**
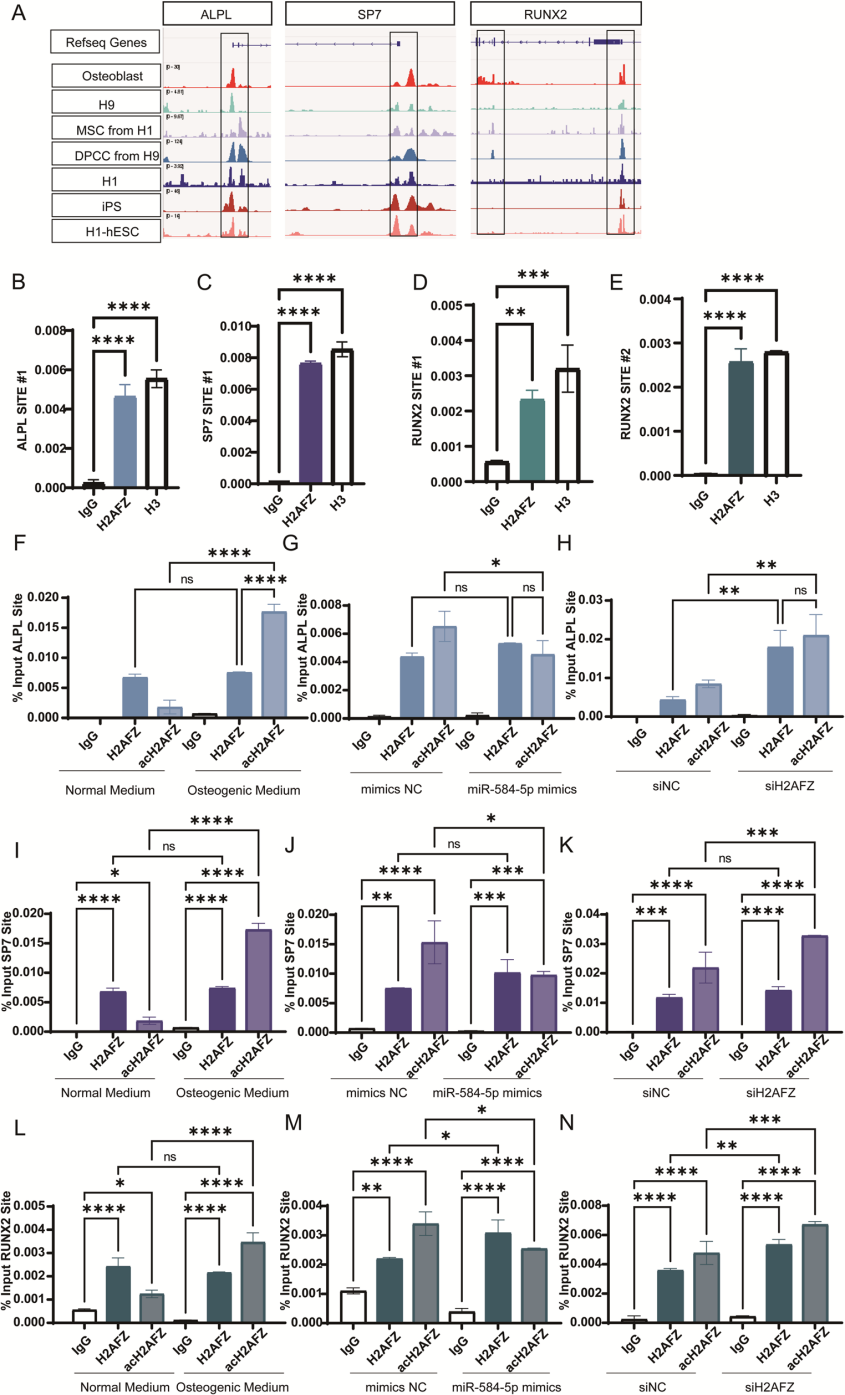
H2AFZ binds to osteogenic gene promoters and modulates their transcription. **A** Integrated Genome Viewer (IGV) analysis of H2AFZ ChIP-seq data showing enrichment peaks at the promoter regions of ALPL, SP7, and RUNX2 in multiple cell lines. **B**–**E** Chromatin immunoprecipitation followed by qPCR (ChIP-qPCR) demonstrating that H2AFZ is enriched at the promoters of ALPL, SP7, and RUNX2 in PDLSCs. **F**, **I**, **L** ChIP-qPCR results showing that osteogenic induction increases the enrichment of acH2AFZ at the promoters of ALPL (**F**), SP7 (**I**), and RUNX2 (**L**). **G**, **J**, **M** Overexpression of miR-584-5p decreases acH2AFZ enrichment at these promoters. **H**, **K**, **N** Knockdown of H2AFZ enhances acH2AFZ binding to the promoters of ALPL (**H**), SP7 (**K**), and RUNX2 (**N**). *p < 0.05, **p < 0.01, ***p < 0.001, ****p < 0.0001; ns nonsignificant

We queried relevant databases and analyzed H2AFZ ChIP-seq data using IGV software, finding that in multiple cell lines, H2AFZ shows enrichment peaks at the promoter regions of ALPL, SP7, and RUNX2 (Fig. 6A). Chromatin immunoprecipitation followed by qPCR (ChIP-qPCR) demonstrated that H2AFZ binds to the promoters of ALPL, SP7, and RUNX2 in PDLSCs (Fig. 6B-E). Osteogenic induction significantly increased the enrichment of acetylated H2AFZ (acH2AFZ) at these promoters (Fig. 6F, I, L). Overexpression of miR-584-5p decreased acH2AFZ enrichment at these promoters, while H2AFZ knockdown increased it (Fig. 6G-N). The acetylation status of H2AFZ influenced its promoter binding and the transcriptional activity of these genes. Through co-immunoprecipitation experiments, we found that HDAC1 can bind to H2AFZ. However, further experiments are needed to determine whether HDAC1 can influence the acetylation level of H2AFZ (Fig. S4A). These data indicate that miR-584-5p inhibits osteogenic differentiation by upregulating H2AFZ, which binds to and represses osteogenic gene promoters.

### MiR-584-5p inhibits osteogenic differentiation by targeting RUNX2

**Fig. 7:**
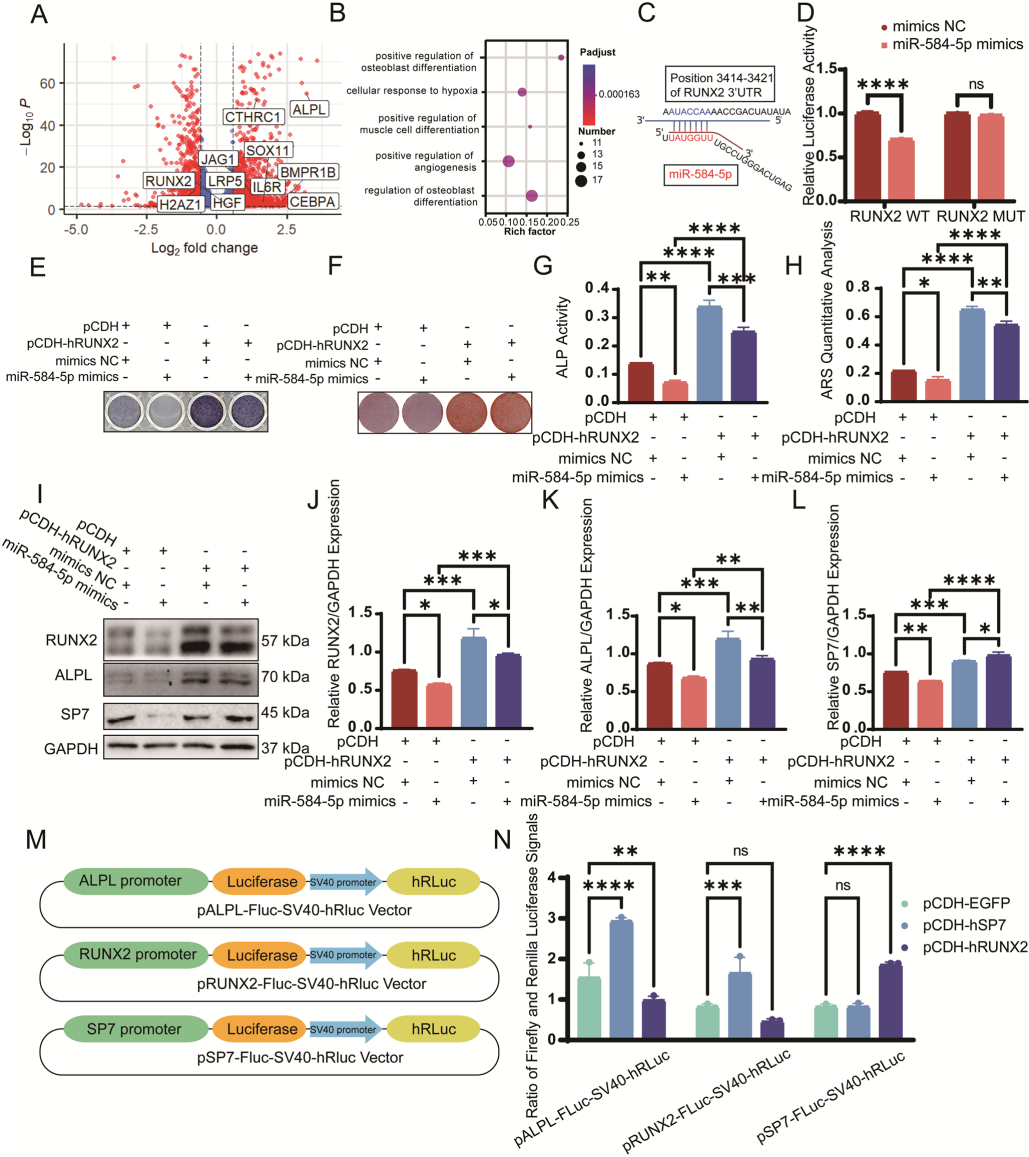
miR-584-5p directly targets RUNX2 and affects osteogenic gene regulation. **A** Volcano plot from RNA sequencing analysis of PDLSCs after H2AFZ knockdown, highlighting significantly upregulated osteogenesis-related genes. **B** Gene Ontology (GO) enrichment analysis indicating involvement of differentially expressed genes in bone development and mineralization. **C** Schematic of the predicted binding site of miR-584-5p in the 3’UTR of RUNX2 mRNA. **D** Luciferase reporter assay showing that miR-584-5p overexpression decreases Luciferase activity of the wild-type RUNX2 3’UTR construct but not the mutant, confirming direct binding. **E**–**H** Functional assays demonstrating that overexpression of RUNX2 rescues the inhibitory effects of miR-584-5p on osteogenesis: ALP staining (**E**), ARS staining (**F**), ALP activity (**G**), and ARS staining quantification (**H**). **I**–**L** Western blot analysis (**I**) and quantification (**J**–**L**) confirming restoration of osteogenic protein levels upon RUNX2 overexpression despite miR-584-5p overexpression. **M** Diagram of dual-luciferase reporter constructs containing 2000 bp promoter regions of ALPL, RUNX2, and SP7. **N** Dual-luciferase reporter assays showing that RUNX2 enhances SP7 promoter activity but not that of ALPL or its own promoter, while SP7 activates the promoters of ALPL and RUNX2. *p < 0.05, **p < 0.01, ***p < 0.001, ****p < 0.0001; ns nonsignificant

To gain further insights into H2AFZ’s role, RNA sequencing (RNA-seq) analysis after H2AFZ knockdown revealed upregulation of osteogenesis-related genes (Fig. 7A). Gene Ontology enrichment indicated involvement in bone development and mineralization processes (Fig. 7B). Gene Set Enrichment Analysis (GSEA) showed activation of the Wnt signaling pathway upon H2AFZ knockdown (Fig. S2), suggesting H2AFZ may suppress osteogenesis by inhibiting this pathway.

Bioinformatic analysis predicted that miR-584-5p targets the 3’ untranslated region (3’UTR) of RUNX2 mRNA (Fig. 7C). Luciferase reporter assays confirmed direct binding of miR-584-5p to the RUNX2 3’UTR, resulting in decreased luciferase activity in the wild-type construct but not in the mutant (Fig. 7D). Overexpression of RUNX2 in miR-584-5p overexpressing PDLSCs restored ALP activity, mineralization, and expression of osteogenic proteins ALPL and SP7 (Fig. 7E-L).

**Fig. 8:**
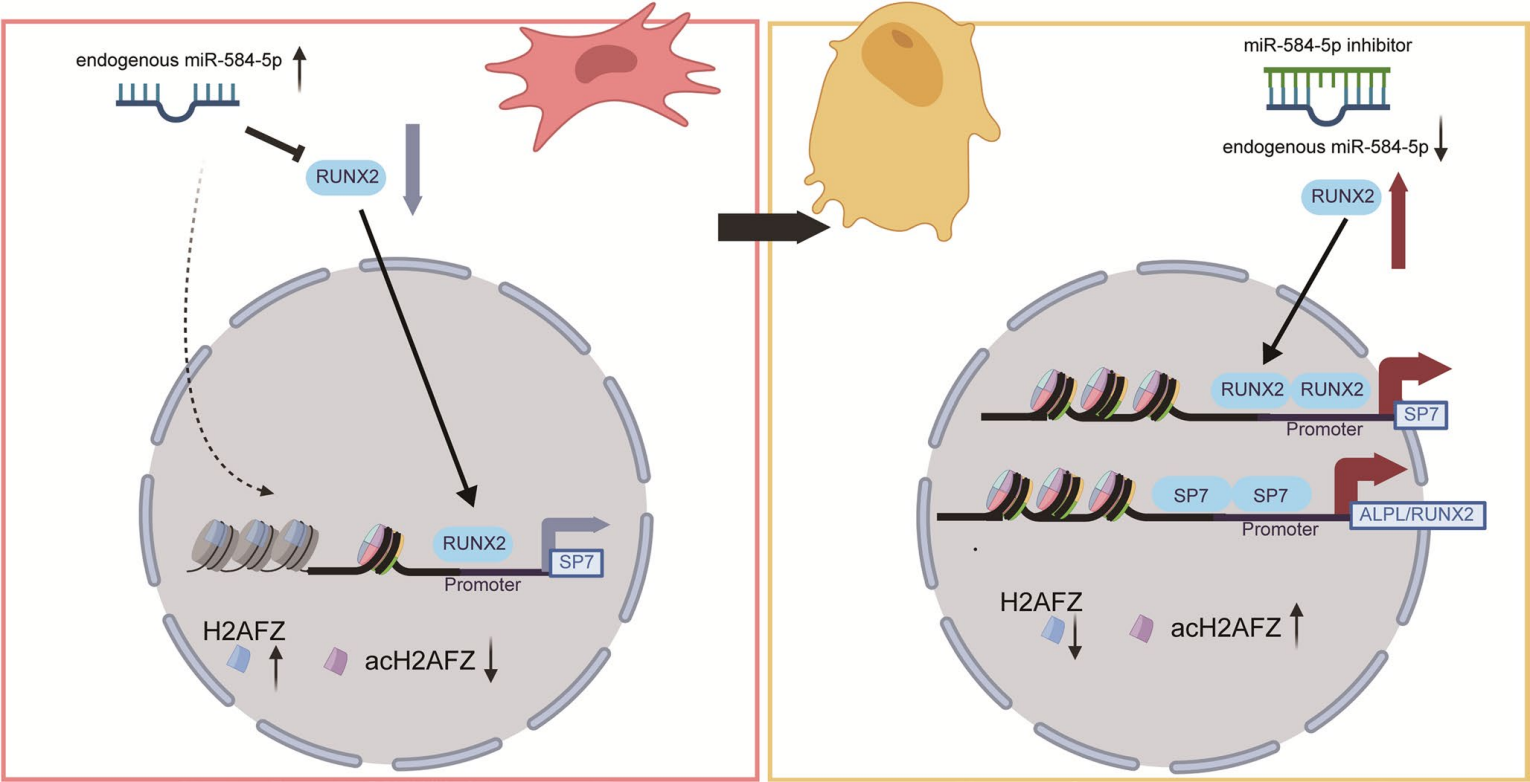
Schematic diagram summarizing the regulatory mechanisms of miR-584-5p in osteogenesis. MiR-584-5p inhibits osteogenic differentiation of PDLSCs by upregulating H2AFZ, which binds to and represses osteogenic gene promoters (ALPL, RUNX2, SP7), and by directly targeting RUNX2 mRNA to reduce its expression. H2AFZ modulates chromatin structure through its acetylation status, influencing gene transcription. RUNX2 and SP7 form a positive feedback loop to promote osteogenesis. The diagram illustrates the dual inhibitory mechanisms of miR-584-5p and highlights potential therapeutic targets for enhancing bone regeneration

Dual-luciferase reporter assays demonstrated that RUNX2 enhances the promoter activity of SP7 but not that of ALPL or its own promoter. Conversely, SP7 activated the promoters of ALPL and RUNX2 but not its own, suggesting a positive feedback loop that promotes osteogenic differentiation (Fig. 7M-N). These findings indicate that miR-584-5p inhibits osteogenesis by directly targeting RUNX2 and upregulating H2AFZ, which together suppress osteogenic gene expression. A schematic diagram summarizes the main findings (Fig. 8).

## Discussion

Our study reveals a novel mechanism by which miR-584-5p inhibits the osteogenic differentiation of PDLSCs. We demonstrated that miR-584-5p acts as a negative regulator through a dual mechanism: upregulating the histone variant H2AFZ and directly targeting RUNX2 mRNA. This dual action represses osteogenic gene expression, highlighting the intricate interplay between epigenetic modifications and post-transcriptional regulation in stem cell differentiation.

The identification of miR-584-5p as a potent inhibitor adds to the growing evidence that microRNAs are crucial modulators of stem cell fate [22]. Previous studies have shown that miRNAs like miR-21 and miR-143-3p influence PDLSC differentiation through various pathways [10–12]. Our findings expand this repertoire by introducing miR-584-5p as a new target for therapeutic intervention in bone biology.

H2AFZ, known for its role in chromatin remodeling and gene regulation [15, 23–25], was identified as a downstream mediator of miR-584-5p. We found that H2AFZ binds to the promoters of key osteogenic genes (ALPL, RUNX2, SP7), and its acetylation status affects this binding and transcriptional activity. Upregulation of H2AFZ by miR-584-5p leads to decreased enrichment of acetylated H2AFZ at these promoters, repressing gene transcription. This aligns with the concept that histone modifications [26] like acetylation are pivotal in regulating gene expression during differentiation [5, 7].

RNA sequencing after H2AFZ knockdown revealed upregulation of osteogenesis-related genes and activation of the Wnt signaling pathway [19, 27, 28], suggesting that H2AFZ may suppress osteogenesis by inhibiting this pathway. Additionally, we identified RUNX2, a master transcription factor in osteogenesis [29–33], as a direct target of miR-584-5p. Luciferase assays confirmed that miR-584-5p binds to the 3’UTR of RUNX2 mRNA, reducing its expression. Overexpression of RUNX2 rescued the inhibitory effects of miR-584-5p, underscoring its critical role.

Our investigation into the interplay between RUNX2 and SP7 revealed that RUNX2 enhances the promoter activity of SP7, while SP7 activates the promoters of ALPL and RUNX2, suggesting a positive feedback loop that amplifies osteogenic gene expression. This regulatory network emphasizes the complexity of transcriptional control in osteogenesis [34, 35].

The therapeutic implications are significant. PDLSCs are promising candidates for regenerative therapies due to their accessibility and osteogenic potential [1]. Understanding the molecular mechanisms that inhibit their differentiation is crucial for developing strategies to enhance bone regeneration. Targeting miR-584-5p or modulating H2AFZ expression could offer new avenues for treating bone defects and periodontal diseases. Moving toward clinical translation, several concrete steps could facilitate the therapeutic application of miR-584-5p inhibitors. Potential delivery systems include exosomes, nanoparticles, and biocompatible hydrogels that could provide controlled release within the periodontal microenvironment [36–38]. Targeted delivery approaches might involve local injection of miR-584-5p inhibitors directly into periodontal ligament tissues following scaling and root planing procedures [39, 40]. Furthermore, combination strategies could incorporate miR-584-5p inhibitors into bone substitute materials during guided bone regeneration (GBR) procedures in periodontal surgery, potentially enhancing the regenerative outcomes. However, several challenges remain, including the development of safe and effective delivery systems, optimization of dosing regimens, and comprehensive evaluation of potential off-target effects before clinical implementation [41]. Given our finding that miR-584-5p knockdown promotes osteogenic differentiation of PDLSCs, an intriguing clinical question arises: do PDLSCs isolated from periodontally diseased teeth exhibit abnormally elevated miR-584-5p expression, which could impair their differentiation capacity? Periodontal disease is characterized by chronic inflammation and altered cellular environments that may dysregulate miRNA expression profiles [42]. Future clinical studies comparing miR-584-5p levels between PDLSCs from healthy and diseased periodontal tissues could provide valuable insights into whether miR-584-5p upregulation contributes to the reduced regenerative potential observed in periodontal disease contexts. Such findings could help explain the compromised healing responses in periodontitis patients and support the therapeutic rationale for miR-584-5p inhibition in clinical applications.

However, our study has Limitations. Our study was Limited to relatively short-term observations, and extending the experimental timeline to 12 weeks or longer could provide deeper insights into the long-term effects of miR-584-5p modulation on PDLSC osteogenic differentiation. Such extended studies would help determine whether miR-584-5p continues to influence late-stage bone maturation processes, including mineralization quality and bone remodeling dynamics, both in vitro and in vivo. MiR-584-5p may have other targets contributing to its inhibitory effects on osteogenesis. Future research should explore these potential targets and delve deeper into how H2AFZ acetylation status affects chromatin structure and gene expression [14]. Investigating the enzymes responsible for H2AFZ acetylation and interactions with other signaling pathways like Wnt/β-catenin may uncover additional mechanisms influencing osteogenesis [43].

## Conclusion

In summary, miR-584-5p inhibits the osteogenic differentiation of PDLSCs through a dual mechanism: upregulating H2AFZ to suppress osteogenic gene transcription and directly targeting RUNX2 mRNA to reduce its expression. These insights deepen our understanding of the epigenetic regulation of bone formation and suggest that targeting miR-584-5p and H2AFZ may offer promising strategies for enhancing bone regeneration.

## Supplementary Information

Below is the link to the electronic supplementary material.

Supplementary Material 1 (XLSX 643 KB)

Supplementary Material 2 (XLSX 71.2 KB)

Supplementary Material 3 (XLSX 22.1 KB)

Supplementary Material 4 (DOCX 154 MB)

Supplementary Material 5 (PDF 13.3 MB)

## Data Availability

The authors confirm that the data supporting the findings of this study are available within the supplemental information. Raw data are available from the corresponding authors C. W. and G. Y. upon request.

## Abbreviations

acH2AFZ: acetylated H2AFZ
ALP: alkaline phosphatase
ALPL: alkaline phosphatase, liver/bone/kidney
ARS: alizarin red S
BMD: bone mineral density
ChIP-qPCR: chromatin immunoprecipitation followed by qPCR
DAB: diaminobenzidine
DAPI: 4′,6-diamidino-2-phenylindole
GAPDH: glyceraldehyde 3-phosphate dehydrogenase
GCN5: general control non-repressed protein 5
GO: gene ontology
GSEA: gene set enrichment analysis
H2AFZ: histone variant H2AFZ
HAT: histone acetyltransferase
HDAC: histone deacetylase
HE: hematoxylin and eosin
miRNA: microRNA
MOI: multiplicity of infection
OCN: osteocalcin
ODM: osteogenic differentiation medium
PDLSCs: periodontal ligament stem cells
PVDF: polyvinylidene difluoride
qPCR: quantitative polymerase chain reaction
RIPA: radioimmunoprecipitation assay
RNA-seq: RNA sequencing
RUNX2: runt-related transcription factor 2
siRNA: small interfering RNA
SP7: transcription factor SP7
TBST: tris-buffered saline with tween-20
TSA: tyramide signal amplification
Wnt: wingless-related integration site
µCT: micro-computed tomography
