# Supplementary figures and images for: Mechanistic insights into miR-584-5p-mediated Inhibition of PDLSCs osteogenic differentiation through H2AFZ upregulation and RUNX2 suppression
Source: Cell Mol Life Sci. 2025 Oct 7;82(1):351. doi: 10.1007/s00018-025-05887-3 (PMC12504162; doi:10.1007/s00018-025-05887-3)

Figure 1H

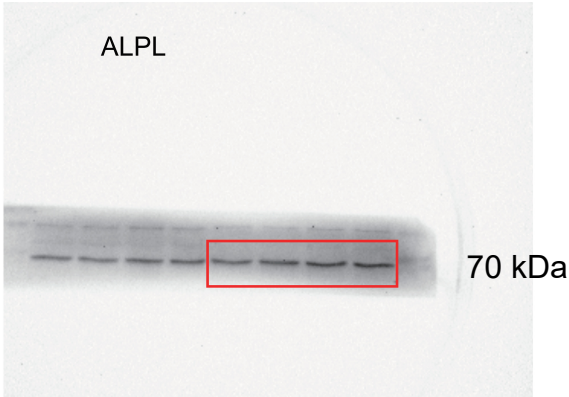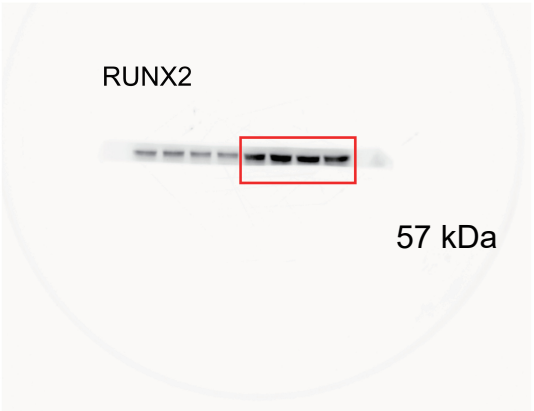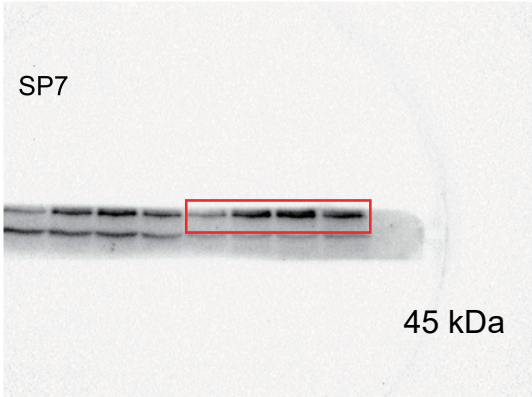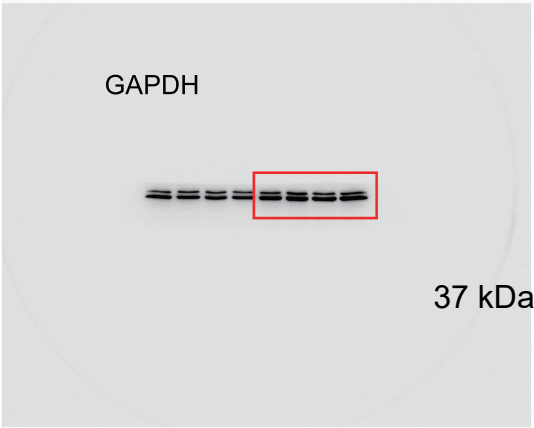

Figure 3B and 3D

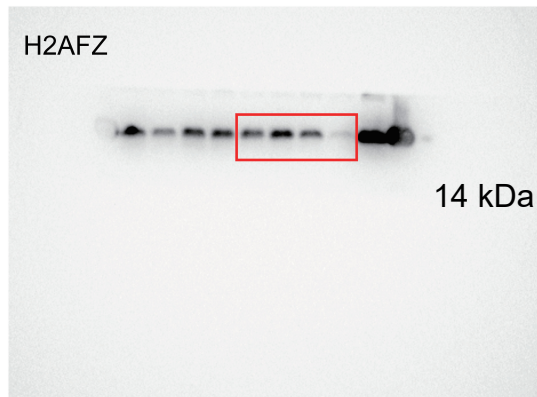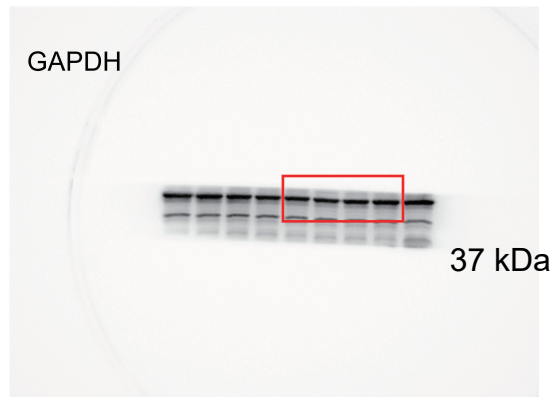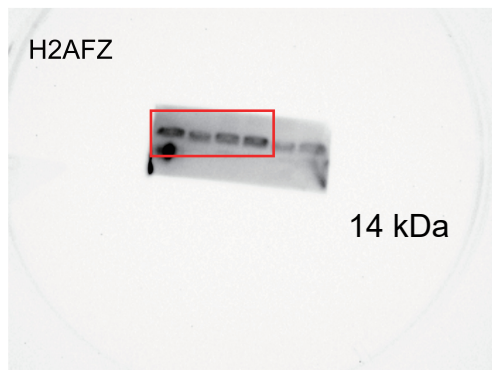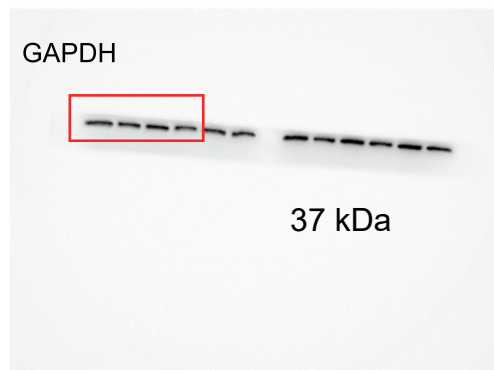

Figure 4B

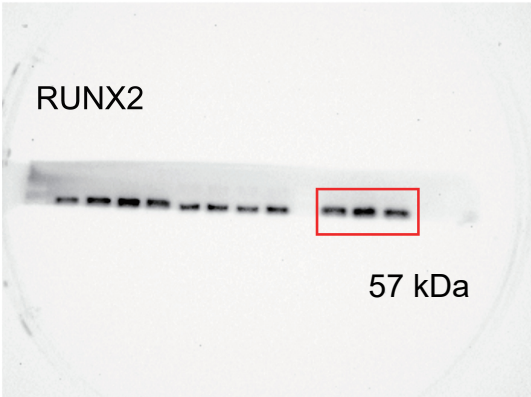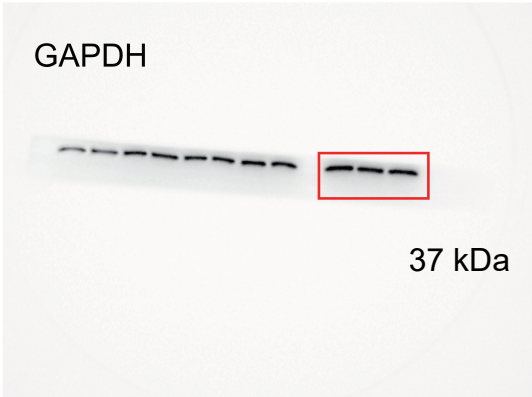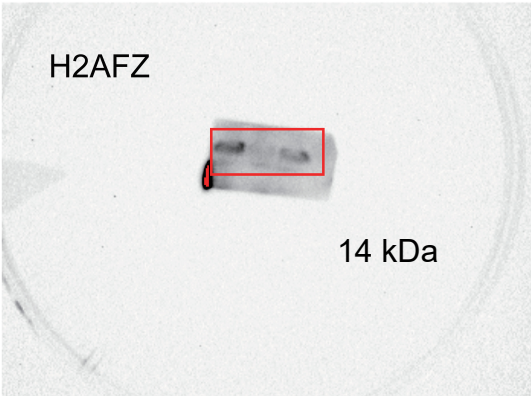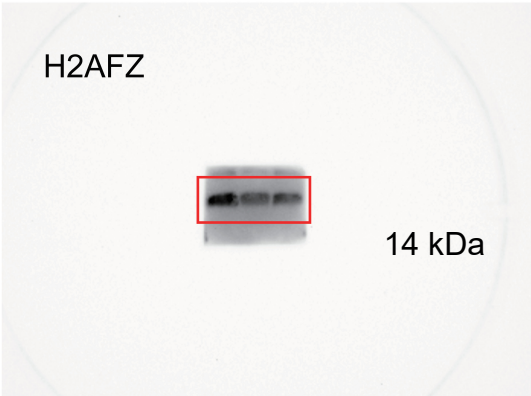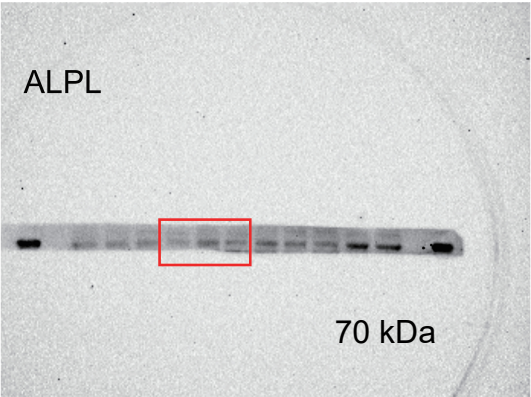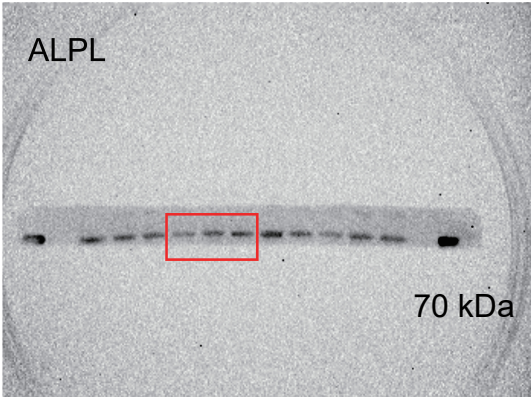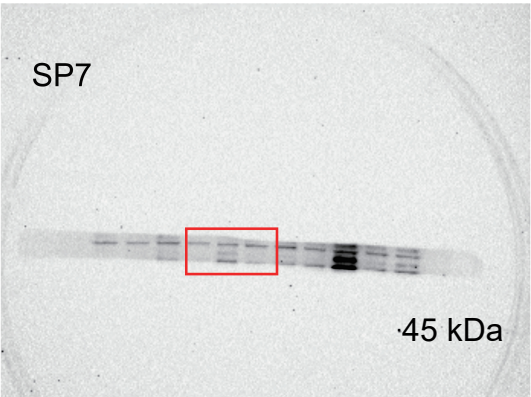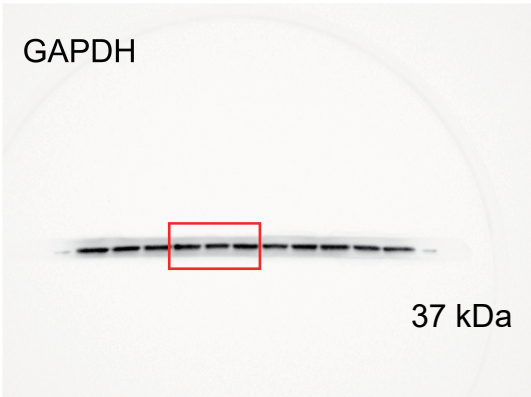

Figure 5A

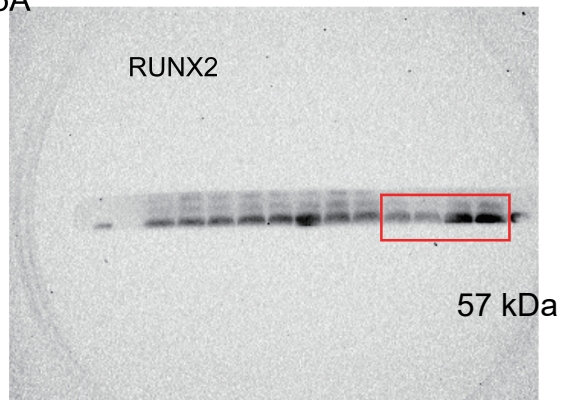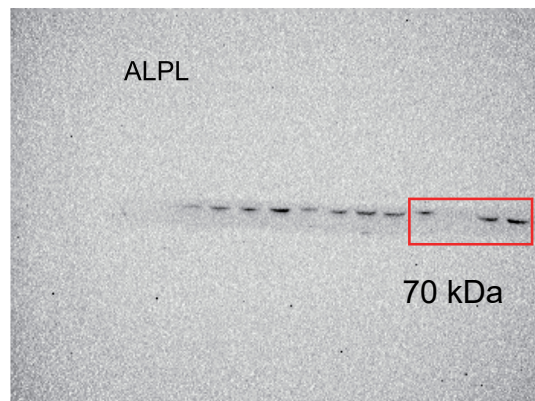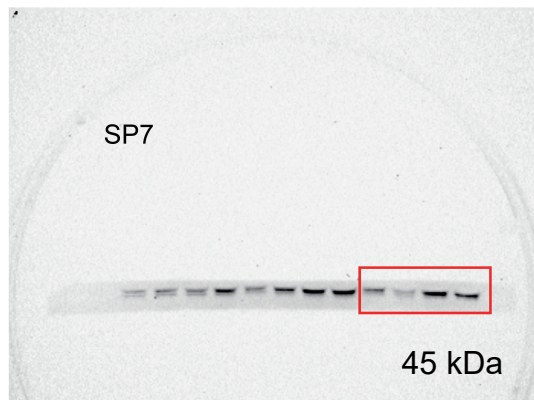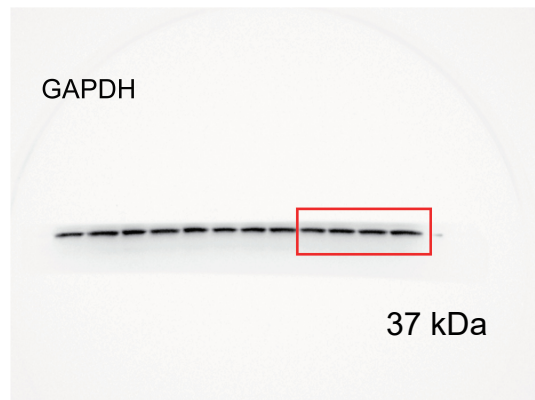

Figure 7I

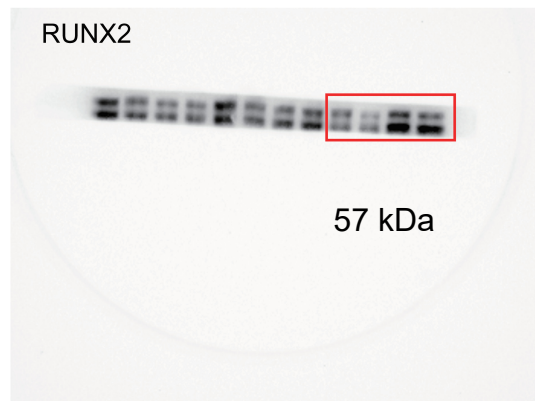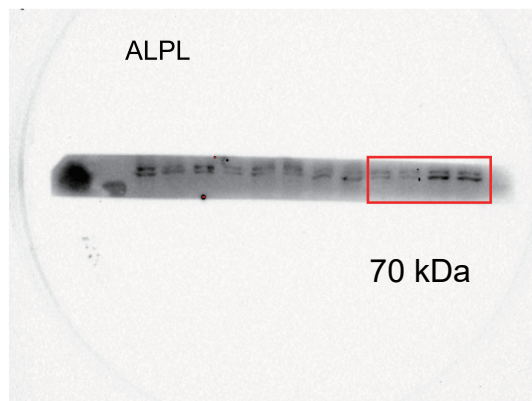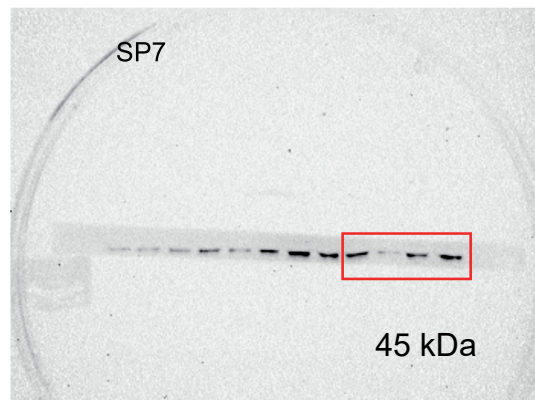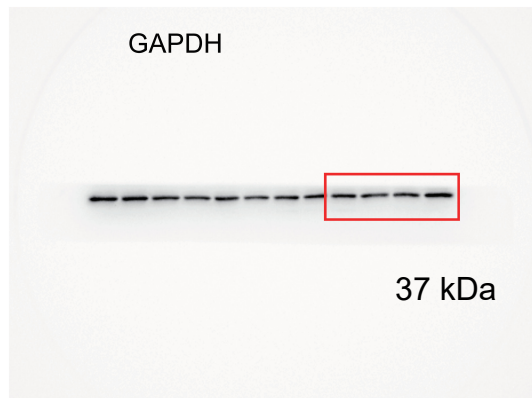

Figure S4A

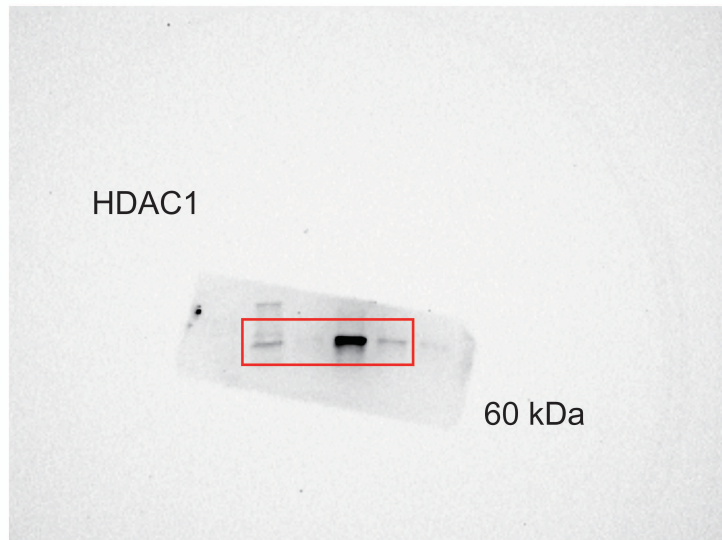

Supplement: Supplementary file 5 — Supplementary Material 5 (PDF 13.3 MB) [file 18_2025_5887_MOESM5_ESM.pdf]
