# Supplementary material for: Mechanistic insights into miR-584-5p-mediated Inhibition of PDLSCs osteogenic differentiation through H2AFZ upregulation and RUNX2 suppression
Source: Cell Mol Life Sci. 2025 Oct 7;82(1):351. doi: 10.1007/s00018-025-05887-3 (PMC12504162; doi:10.1007/s00018-025-05887-3)
Supplement: Supplementary file 4 — Supplementary Material 4 (DOCX 154 MB) [file 18_2025_5887_MOESM4_ESM.docx]

**Table S1. Constructed sequences used in this study**

| Name | Sequence |
| --- | --- |
| miR-584-5p mimics | UUAUGGUUUGCCUGGGACUGAG |
| mimics NC | UUGUACUACACAAAAGUACUG |
| inhibitor NC | CAGUACUUUUGUGUAGUACAA |
| miR-584-5p inhibitor | GAGUCAGGGUCCGUUUGGUAUU |
| siNC | CAUGUCAUGUGUCACAUCU |
| siH2AFZ-1 | GUAGUGGGUUUUGAUUGAG |
| siH2AFZ-2 | AAAGGACAACAGAAGACUG |

**Table S2. Primers for RT-qPCR**

| Genes | Forward primer 5’-3’ | Reverse primer 5’-3’ |
| --- | --- | --- |
| *ALPL* | AGACGCAGAAGGAAGATGCC | ACTCTCGGTCTCTCTGCCTC |
| *SP7* | TTCTGCGGCAAGAGGTTCACTC | GTGTTTGCTCAGGTGGTCGCTT |
| *OCN* | CGCTACCTGTATCAATGGCTGG | CTCCTGAAAGCCGATGTGGTCA |
| *RUNX2* | CCCAGTATGAGAGTAGGTGTCC | GGGTAAGACTGGTCATAGGACC |
| *GAPDH* | GTCTCCTCTGACTTCAACAGCG | ACCACCCTGTTGCTGTAGCCAA |
| *H2AFZ* | GCAACTTGCTATTCGTGGAGATG | CAGGCATCCTTTAGACAGTCTTC |
| ALPL SITE#1 | TGGGCAGAGACGGATAAAGA | GGCTCTGACCTGCTCCTTG |
| SP7 SITE#1 | GAGACTGGAACGCACTGACA | TGCGTCACTGTGTGCATTTG |
| RUNX2 SITE#1 | CGCCTCACAAACAACCACAG | GACTCTGTTGGTCTCGGTGG |
| RUNX2 SITE#2 | CCCCATTCCAAGCTGCAAAC | GCAGATCTTTGCCGGAGTCT |
| U6 | CTCGCTTCGGCAGCACA | AACGCTTCACGAATTTGCGT |
| miRNA-584-5p | GCTTATGGTTTGCCTGGG | GTGCAGGGTCCGAGGT |

**Table S3. Constructed sequence of Plasmids**

| Plasmids | Sequence |
| --- | --- |
| WT RUNX2 | TGCAGTACTTTGAAATATATCAGCCAAAACCATAACTTACAATAATTTCTTAGGTATTCT |
| MUT RUNX2 | TGCAGTACTTTGAAATATATCAGCCAATTGGTATACTTACAATAATTTCTTAGGTATTCT |
| pALPL(human)-Fluc-hRluc-Neo | AAAATTAGCCGGGCGTGGTGGTGCACACCTGTAGTCCTGGCTGCTGGGGAGGCTGAGGTGGGAAGATCACTTGAGCCCGGGAGGTCGAGGCTGCAGTGAGCTGACATCATGCTACTGCATTCCAGCCTAGGCAATAGAGTGGGACCCTGTCTCAAAAAAAAAAAAAAAAAAAAGTCAGGGGTGCTGGCCCCATGATAGGTGCAATGGGTGCCTCCAATTCCCTCTGGCTCTGCCTCCCAGCCTCTGTCCAAGCAACAGGCAGATTTTCCATGCCTGGGGACTTGCCCCTGGCTCACTGATGATGATACCATCTTAAGTCTCCTGGAATCCTTAAACCCTTCCTTGGCATTTGTGAGCAAGTATTGAGCCCCTCCCATGTTTTAGGCCCAGTGCTGGGTGCTTTCACCTGCAATTTCTTACAGAGGATGGTATTTCCTAAACTCCATTCATTTGCTACCTCCATGATTTTTGCCATATCACCCAACCCATGAAATACATAAATAAATAGTATTTACTCAATATTTTGACTCACTTTTAAAAAACATAAACTTATCTGAAAAGGGAACCTATGTCACGCCTCTAAATAAACAGTATCAACCAAAAATACAAAAGAAACAAAAATAAATACAAAGACATTCTTGGTTTCTGGCACCAGGCCCCAGTTCTGGCTCCAACCTTACTGAGAAGTCACATGATCTCTCTGGGCCTCAGTTTTCTCCTCTGGAAAATGGAGCTTTTGGAAGTTAATGCATGCACAGTGCCTGGCCCTGAGACTGGCATGGAGTGAGTGGAAGAGAGGTTGCCTGACCTGGCTAAAATTGGTTCTCTGGGCAGACATTTTCCCAAGGGCCACTGAGAAGACCCTCCTGTTAGGAGTCAGTGAGCTCTGTCGCTGGAGGCATTCAAACAGAGCCTGCGGACTTCTCACTGCGAAGTTGCCCAGAGAATTCAGTGCTCAGAGGAAAGGGAGTGGTTATTCCATCAGAGCTGGTTCCCCAGGAGCGGGAGCAGGGCCTGTAGCACCCAGCCTCTGTCCCTGGCTCCCGTCTATCCGGGATTTTAGCGTTTCCTCTGTAGTTTTCAAGCACTGTCTCATATGACTCTCGCACCAGCGAGAGGCCAGGGGAGATGGTGTCTGCCTGTTAAAGAGGGGCAGGCTGGTCCACATAGGTCAAGTGACTTGGCCAAGGTCACCAGAGCAGAGTTTTGAACTTGAGCTGTCTGACTCAACTGCCTGGGAAGTGCCTGCCCCTCCTCTGGCATCCAGGGAGCATGTCCTGGGGCTCTGGCTGGGACATAGCCGGACACCTGCGGGCCCTTTACGTCTCTAAAGAGAGAAAGAGGGAAGGGCCCCTGTCTAGGGGGTGGTTTCCCTCCAGATGCCACCCCTCCGAGGTCCCCTTCTGCTTCTTCTTGCGGTAGCCAGGGAGGCAGCCCACGGGCAGGGAAGCGGGGGTGGGGGTGCAGAGTCAGAGGTGCACGTGGACAGAGACAGAGAGACAGGGACACGTGGGCAGAGACGGATAAAGACAGAGACCCAGAGAAAGCCAGATATGTTGACAGACACAGAGACAGACGCCAGAGAGGAAGGCAGACAAAGAGACGGGTGGAGACAAAGACTCCCACCAAGAGACGCAGAAGGAAGATGCCGACGGTAAAGACAAAACAGGAGACGCGCGCAAGGAGCAGGTCAGAGCCCAGGCTCGCTGAGAGAGGAAGGGCTGGGCTGGGGCAGCCCGGAGGCAGAGAGACCGAGAGTGCGGGGCGGGCGAGGGACGCCAGGGCCGCGTCACCCCAGCCCGTTCCTAGCTCCGCTCCCGGCAGGGGGCGCCCTGGCCTCGTGGCACGACCGGCCCGCGGGGCGCGGGGCTCGGGCCGGGGGCGGGGCCGGGGCCGGGCTGGGGAGGGGTTGGGGCCGGGGGCGGGGGAGGGGGCGGGCTGCCCGGGCCTCACTCGGGCCCCGCGGCCGCCTTTATAAGGCGGCGGGGGTGGTGGCCCGG |
| pRUNX2(human)-Fluc-hRluc-Neo | GGGAGGGCGTGAGAGATGACCCAACCATCTTTCTTCCTTGATGCTTCGAGGGGGAGGCATTCTAATTCTTCATTTATTTTATAAAATCACTTGTTACTGTGTCAGCTACTCCACTAGGAATCTTTGAAACTTTGGCAACTGATGATTTTTTTCGCAAACACGTTTTCAAGCTTTGGAAGGCTTTAAAGAGTTTGGTAATAAGCAAGAAACGTAGTAGTACACAACGCCGAGGGTAAAACGGCCCTGTGCCCCCTTCTTGGGGTGTAATAGCCTCAAGCGGATTTCCCGGCTTCTGCGGGCGCAGCACATTGTTTTATTTGTTTTGAAGGCTCAGAGTTTGAGGCTGGTCGTAGACACCCACGTGCTCTGACTCTCATCAGCGTAATGATCGACTTAGCCAGAGTCGTGTCTATATAAACCACAAAAACCTAATCATTAGAAATCCCAGCCTCCAAAAACCACATTTTAGGTAAAAAGTGCCGCTTTTTTTCCGCGCTCCTTCATCCTCTCGACCACAACTTTTTGGGGGATCCAAAGTCGCTTTTTAAACCCACACTAGGAAATAAATACATAAATAAACAGCCACGGAAGCATTTTGAATTTTTTAGTTCGTATTTTTAATTTTTCCCCGTGAGCGCTGGGGAAGACAGACACGAAACTTAGTCTTAGATGTAGAATAGACTTAATAAAATATTTAAATTCATGGAAAACGGCTGGTGCTTTTCCCCAGCAAGGGTTTTGCAACTTATTTTAATCACAAAGCTGACAGTTTTTCAGTCCTTAAAACAAAAACAAACAAACAAACACCTCATTCTTGTTCTCTAGGAATGACAAGATTGAATATATAAGAAAAAAGAAATGTCCAGCTATGCACTTGAGTTCCTAAAAACTATTTTGACTAGATCTCCCTCCACCCCCCCTCCTCATTTTTTTTGTGTCACTTAAAACAAAAAACACCCAAACCCTGCAAAATCTTTTTCCCTTGGGAGTCGCTCAGACAGAGGTGGGGGTAGAGGTGGGAGGGTGAAGAGTGCTGAGAAGTCAGCACAGAGGCTCAAGAATTTTCTTTCCTCCTCTCCTGAAGTTACAACGAAAAATAGCGACAGTCAGATCAGCCGGGGGCTCTCTGGTGTCTCGGCTTCAATCTCTTCCTACAAAGAGGGGGTTCCTTGTCCTAGGGGTGGGGAGAGCAAGAGGACGCGACCTGGCGAGATTGCCCCTAGTCTGGGGCCGCCTCTAGGCTTCGGAAACTGCACACGGCGCGCGGGGGGCTCCTTCTCGCGGCCGGCTGGCGGCGGCAGCGGCTGCGATTCGCAGGCTCGAGATTCGTTGCCCCGAGATCCGCTGCCCACGACTTACCTCTTGGCACCTTGAAGCGCGAGAGACAGGGGAGCTTTGCAAATTGCAGGAGGGAGTGGATGCAAAGAGGTGGCTGTGAGAGGGCGAGAAGAAAAGAAGGGAAAGGAAGGAGCCTAGCCGGGGAGCCGGGCTGGCAGCCGCCGCGGGAGGAATCTGAGCGTGCAGCCCGCGCGGGGGGAGCGGCGGCCGCCGCGGAGGCGGCGGCGCGGGAGGGCGGAGGCGCGGCCGCCTGCAGCTGGGTAGCAGCGCGCGGCGCGGCGCGGCGGTGGCGGGCAGCGCGCGGCAATGCCGGCCCCGGGGCTGGACTGCTGAACCCACACCGCTTCACCACCGGACTCCAACTGGGAGGGGGCGCCAGCGCACCCAGGACGCGGTGCCCCAAGGGACCCCATTCCAAGCTGCAAACTCAAGTCCCCCGCCTCCCCAGGCCAAACACACCCCCGCGCCCGTTCGCAGCTGCCACCCCGGGTGTTCCAAAGACTCCGGCAAAGATCTGCGCCTCCCGGGTCTCCCTACCCGCCAGCCCGACTGCGCCGCATCGCCAGACTCTGTTGGCCCATCAGACTCCGCCCGGCAGTCGGCCTCATCAAACTTGATTTCTCACCTCCTCAGCCCCATCACCTCCATCCTCTTTCCCCCC |
| pSP7(human)-Fluc-SV40-hRluc-Neo | CCTCAGTGTCCCGGCGCGGCCGAGGGGCGGGCGGCAGGGGGCGGGGCGCGGGCGGGGCCGGCCGGGCCAGGCGCCCGGGCAGGAGGCAGCTGCATATCTGCGGTCCCGACCACAATTGCTGGGAGTCGCGTCCCGGAGACTGGCGGCGCTGACTCTGCCCGCTGCGGCCGCCGCGCCGATTAGAGCCCGAGAGGGAGCGCCAGCCCGGGGGAGGGGGCGGCGGCGGGGGAACTCCGGATGCCCCTTCGCCTCCGCCCAGCCAGCGCCCCCCGCTGCCCGGCCCAGTCCTTTCTCAGCCGCCCGAACCCAGGACCGGAGTCCTGCCTCTTCCGCCGTAGCTTTCTCGAGGCTGCATCCAAGCTTCTTCCTCCGGATCTTGCAGTGCCCGAGGTCCCGACGGTGCCAACATCCCGTTGGCCCCCAGCCTGCCAGTCTCTCATGCCCTTTCTCTTCCCCATCACCCCACACAGAGAGGAGTAGGGATCTCAGAGCTCCCCCATTCTCTTATCCTCCGCTCCCCAATGCACTCTCTAAGCAAAAGCCGTTCCAAGCGAAGAAGAGAATCAGAGACTCAGAGGACGGGCTTTCCAGGGCCGGAGCACCCCCTGCTGGCTGGCCCGAAGCCCCTCCCCCTCCCCCTCCAGCTGTGACCTCCAGCTGTGGTGGCTGGGAAGAGCTGGCCCCTTTGATCTAGGAGCGTGGCAACCCCCTCCAGGCGCCCCATTAACCTCCATTATCCCAATTAGGCAGCCCTCCCAGTCCTGGATCCAAGCTTCAAAGCCCCCTCAGTTGGGAGAAAAGGGAGCTGAATCGTATGCCCCCTACCTCCCCACCAAGCCACACCACTTTCAGACAAGACCCAGGTGCTCTGGTCTCCAGCTTGTGTACATGATAGCAGGGCCAGAAGAGGGACCCTCAGGGGCCACCCTCGTCCCAATACTGAAACTTCATTAAAAAGAGAAGCAGATAGGCAAGGGGACCCTCTTTTGCTGGCCCATTTTCCTGCATTCAGTGATTTCAGTGGCAGCATCCAGGTTCCTGGCTGACCTGAGCTTCCCATCAACTTATCCAGCTTTATCCTGTGGAAAGGGGAGCCAAGAACCAACCCACTCCTCCCTTAATCCTATCTCAACACTCCCTTCCTCCCTCCCTCCTGGAACAATTTCCGCTGGGGGAGCCAGAGCAGGGCCCAGCACAGTGGCCGCTTCCTGGAACATTTTCCTGTATCCGAAACCGCGGCTCCCTCCCTAGGGACCGGCTGATCAAGGCCAGATGGGAGAAGGGATGGGGGAGGAGGAGAGGGAGCCAGGCTCTCACCATCACCCCCTCCCAACACATAGGCACACCACAGGAGCCCCTGCCCTGACACACAGAGGCTGACACAGGCACACACCACATGTCATAGTCACACCATGGGCATTCATGGGGACACATGAACCACAGAGGCCAGGTCTTAGAAGAAGTTAACACACCCGTAGATGATCACCCTCTCTCTCTACCTGGCCCTCTAAAGGACCCTAAGGCCTTTCTTGCCAGATAAGGAGACCTTAGGAATCCAAGAACATTGGCACAGGATAGCAGAAGGACCTCAATTAACAGAGGAGACAGACAGAGACATCTAATGTGCCCTGCCAGACAACACCCACCAACCATCCCATTCCAATTCTAGATGGCACTCAGGCCTGCCTAACCCATCATCTCTCCAGGATCCTGGGAACTGGGGTAGAGGAAGGCTGTGGGAGCTTCTTCTCAAGTGCTACCTTCAGAGCACTTTCTAGTTGCTTCCAGATTCTTGGAGAAGATGCATATGATGTCTAGTGGGCTCGCTCATGGCAGCCAAGGGACAAAGAAAGGGGAAGAATGTGTGCAGCTGCAGGGAGCTCTGGGAGCCTAGAGGAGGAAGGAGAACACCAGAGTAACTCCTGGAGACAGAGAGTAACCCTGGGGATTTTGTGGCCTTACTGGTGACATACTGCCCCAGCACTGCTCCTCCTGATCTC |

**Fig. S1** H2AFZ knockdown promotes bone regeneration in a rat calvarial defect model. **A** Micro-CT images show three-dimensional reconstructed images of the rat calvarial defect models in different groups. Micro-CT images analysis displaying enhanced new bone formation in defects treated with H2AFZ-silenced PDLSCs compared to controls. **B**, **C**, **D** Quantitative analysis of bone mineral density (BMD) (**B**) bone volume fraction (**C**) and showing significant increases in the H2AFZ knockdown group. **E** HE and Masson's staining of calvarial sections revealing increased collagen deposition and new bone formation in the H2AFZ-silenced group. **F**, **G** Immunohistochemistry (**F**) and immunofluorescence staining (**G**) of calvarial defect tissues showing increased expression of the osteogenic marker SP7 and reduced H2AFZ expression in the H2AFZ-silenced group versus control. *p < 0.05, **p < 0.01, ***p < 0.001, ****p < 0.0001; ns nonsignificant

**Fig. S2** H2AFZ knockdown activates the Wnt signaling pathway in PDLSCs. **A** Gene Set Enrichment Analysis (GSEA) plot showing significant enrichment of Wnt signaling pathway genes in PDLSCs after H2AFZ knockdown. **B** Heatmap of differentially expressed genes related to osteogenic differentiation, indicating upregulation upon H2AFZ silencing.

**Fig. S3** Bioinformatics analysis of miR-584-5p target genes and validation experiments. A Bar chart showing the number of target genes predicted by different databases (TarBase, DIANA-microT, Pita, miRanda, miRtarbase, MicroCosm, and miRdb) for miR-584-5p. B Protein-protein interaction network analysis displaying predicted interactions between miR-584-5p target genes, with RUNX2 as a hub gene. C Gene Ontology (GO) molecular function enrichment analysis of miR-584-5p target genes, showing significant enrichment in DNA-binding transcription activator activity, histone H2A kinase activity, and other regulatory functions. D Bar chart quantifying the number of genes related to bone keywords among miR-584-5p predicted targets, demonstrating potential involvement in osteogenic processes.

**Fig. S4** Co-immunoprecipitation validation and dose-response analysis of miR-584-5p inhibitor. A Co-immunoprecipitation experiment showing the interaction between HDAC1 and H2AFZ proteins. Input, IgG control, HDAC1, and H2AFZ immunoprecipitation conditions are shown. B Quantitative analysis of cell viability in PDLSCs treated with different concentrations of miR-584-5p inhibitor (0-100 nM) compared to inhibitor negative control. C ALP staining images of PDLSCs treated with increasing concentrations (0-100 nM) of miR-584-5p inhibitor or negative control, demonstrating dose-dependent enhancement of osteogenic differentiation. D Quantitative analysis of ALP activity, confirming significant dose-dependent increases in ALP activity with miR-584-5p inhibitor treatment. *p < 0.05, ***p < 0.001, ****p < 0.0001; ns nonsignificant

**Fig. S5** Characterization of isolated human PDLSCs demonstrates multipotent differentiation capacity. **A** Primary PDLSCs migrating out from tissue explants, showing typical cellular morphology. **B-F** Multi-lineage differentiation capability of PDLSCs: osteogenic differentiation (Alizarin Red staining, B), chondrogenic differentiation (Alcian Blue staining, C), colony formation assay (D), adipogenic differentiation (Oil Red O staining, E), and ALP staining (F). **G-I** Flow cytometry analysis confirming the expression profile of isolated PDLSCs: positive for mesenchymal stem cell markers CD90, CD73, CD44, and CD105, while negative for hematopoietic cell markers CD11b, CD19, CD34, CD45, and HLA-DR (FL2-H) (FL1-H = isotype control), validating the stemness and purity of the isolated cell population.
